# Cord blood innate-like T cell responses in neonates born to healthy women and women living with HIV

**DOI:** 10.3389/fimmu.2025.1628145

**Published:** 2025-08-22

**Authors:** David Rach, Hao-Ting Hsu, Nginache Nampota-Nkomba, Godfrey Mvula, Felix A. Mkandawire, Osward M. Nyirenda, Bernadette Hritzo, Francesca Boldrin, Giulia Degiacomi, Laura Cioetto Mazzabò, Riccardo Manganelli, Andrea G. Buchwald, Franklin R. Toapanta, Marcelo B. Sztein, Miriam K. Laufer, Kirsten E. Lyke, Cristiana Cairo

**Affiliations:** ^1^ Molecular Microbiology and Immunology Graduate Program, University of Maryland School of Medicine, Baltimore, MD, United States; ^2^ Institute of Human Virology, University of Maryland School of Medicine, Baltimore, MD, United States; ^3^ Blantyre Malaria Project, Kamuzu University of Health Sciences, Blantyre, Malawi; ^4^ Graduate Program in Life Sciences, University of Maryland School of Medicine, Baltimore, MD, United States; ^5^ Department of Molecular Medicine, University of Padova, Padova, Italy; ^6^ Center for Vaccine Development and Global Health, University of Maryland School of Medicine, Baltimore, MD, United States; ^7^ Department of Microbiology and Immunology, University of Maryland School of Medicine, Baltimore, MD, United States

**Keywords:** spectral flow cytometry, HIV-exposed uninfected (HEU) infants, gamma delta (gammadelta) T cells, MAIT (mucosal-associated invariant T) cell, NKT (natural killer T) cell, Malawi, intracellular cytokine staining, cord blood (CB)

## Abstract

Innate-like T cells (ILT), including γδ T cells (Vδ2s), Natural Killer T cells (NKTs) and Mucosal-associated Invariant T cells (MAITs), integrate innate and adaptive immunity, playing important roles in homeostatic conditions as well as during infection or inflammation. ILT are present on both sides of the fetal-maternal interface, but our knowledge of their phenotypical and functional features in neonates is limited. Using spectral flow cytometry we characterized cord blood ILT in neonates born to healthy women and women living with HIV. We describe extensive phenotypic and functional heterogeneity within the cord Vδ2 cells at baseline and following activation. In neonates born to women with HIV, we observed modest differences in ILT frequencies ex-vivo and altered proportions of Vδ2 cells producing IFNγ+ or TNFα+, both ex-vivo and after expansion, compared to HIV unexposed infants. Consistent with prior studies, infants born to mothers who initiated ART before pregnancy exhibited less immune perturbation overall. Herein we expand our knowledge of ILT at the maternal-fetal interface by a comprehensive phenotypic analysis of these rare subsets.

## Introduction

Innate-like T cells (ILT), including Vγ9Vδ2 T cells (Vδ2s), Natural Killer T cells (NKTs) and Mucosal-associated Invariant T cells (MAITs), integrate innate and adaptive immunological functions in homeostasis and host defense. After T cell Receptor (TCR)-dependent or TCR-independent activation (mediated by cell-subset specific ligands and cytokines released by innate cells, respectively) (1–3), ILT rapidly mount an inflammatory response in the form of Th1-like cytokines and cytotoxic granule release (4, 5). ILT are present on both sides of the fetal-maternal interface during uncomplicated pregnancies (6–8). Maternal NKTs and MAITs increase in the placenta during the course of pregnancy and produce Th1-like cytokines upon stimulation. Cord blood ILT predominantly express Th1-like cytokines, while cord Vδ2s and NKTs can also produce IL-17A in response to *in vitro* antigen stimulation (7, 9–11).

In uncomplicated full-term pregnancies, several mechanisms converge to create and maintain fetal-maternal tolerance (12). However, maternal infections during pregnancy have the potential to perturb the unique immunologic microenvironment at the fetal-maternal interface (13). This can disrupt immunologic homeostasis, as infections may cause activation of immune cell subsets (including ILT) triggering inflammation. Additionally, pre-natal exposure to maternal infections and inflammation may impact the developing fetal immune system. In mice, IL-6 production in response to prenatal exposure to maternal *Yersinia pseudotuberculosis* led to amplified Th17 responses to infection later in life (14). Studies of human infants with prenatal exposure to pathogens, including *Plasmodium* spp. and Hepatitis C (15–17), reported increased Th1 responses to infection and immunization in early life.

In particular, several studies observed a higher infectious morbidity (due to enteric and respiratory infections) in infants exposed to human immunodeficiency virus (HIV) *in utero* but born uninfected (HEU) compared to their HIV unexposed (HU) counterparts from the same communities, especially during the first six months of life (18, 19). Multiple investigations have described underlying immunological defects in HEU infants, with results differing across studies likely due to differences in maternal clinical characteristics, including anti-retroviral therapy (ART) regimens, or magnitude of viral load, and infant feeding modality (20–29).

Our knowledge of fetal ILT subsets, either during a healthy, uncomplicated pregnancy or following immune perturbation during gestation, is limited. The placental environment of pregnant women living with HIV displays inflammatory features, influenced by the peak and duration of viral load (VL) and/or maternal immune activation (30–33). Moreover, the use of ART is widespread and recent studies suggest that certain antiretrovirals may impact the mitochondrial metabolism in a way which alters the immune system and contributes to the inflammatory placental microenvironment (34–36). To our knowledge, no other study has analyzed cord blood ILTs in HEU neonates, despite the possibility of HIV/ART prenatal exposure resulting in a perturbation of these subsets. Two studies on 8- and 24-month-old infants included limited information on total γδ T cells, with minimal assessment of their heterogeneity and function (21, 27). Prior technical limitations related to the use of conventional flow cytometry (CFC) (a limited number of markers that can be assessed simultaneously) and mass cytometry (>40 markers per cell but slow acquisition rate, often limiting depth of analysis) have prevented accurate profiling of rare neonatal ILT subsets and exacerbated the challenge of discriminating differences due to prenatal exposure to pathogens from heterogeneity in individuals in clinical cohorts. This may be particularly relevant for prenatal HIV exposure where ILT perturbation due to metabolic milieu (rather than direct antigen stimulation) may be subtle. Spectral flow cytometry (SFC), with its capacity to resolve more fluorophores than CFC (based on the analysis of the entire emission spectrum) and the ability to rapidly acquire a large number of cellular events, enables comprehensive phenotypic and functional analyses of rare cell populations and thus provides a platform to deepen our understanding of ILT cell subsets at birth.

To this end, we developed a 29-color SFC panel which includes lineage, activation, and differentiation markers as well as cytokines, to analyze ILT cells in a cohort of Malawian infants, comparing neonates born to mothers with and without HIV infection. Consistent with prior studies for other cell subsets, we observed modest differences in ILT frequencies and cytokine production between unexposed and HEU infants, with this subtle perturbation mostly affecting neonates born to women who started ART during pregnancy and had elevated viral load at enrollment.

## Materials and methods

### Participant recruitment and study design

As part of an observational longitudinal cohort study designed to examine clinical and immunologic impact of exposure to maternal HIV, pregnant women were recruited during their first antenatal care visit at two health centers in Malawi, the Ndirande research clinic in Blantyre, an urban center, and the Bvumbwe Health center in Thyolo district, a rural area. HIV prevalence during antenatal care (ANC) was estimated to be 12-14% for both sites in 2023 (37, 38). The study was approved by the Institutional Review Board of the University of Maryland, School of Medicine and the Research Ethics Committee of the Kamuzu University of Health Sciences. Written informed consent was obtained from participants before screening and study procedures.

Our cohorts were defined on the basis of maternal clinical ART history and HIV viral load (VL), and stratified as follows: a) newborns of HIV uninfected women (HU); b) newborns of women living with HIV who began ART before conception and had an undetectable VL through pregnancy (HEU-lo); and c) newborns of women diagnosed with HIV infection and high VL (>1x10^4^ copies/ml) at >20 weeks of gestation (HEU-hi). VL was determined for pregnant women at enrollment and repeated at delivery with CD4 counts. Infants who were found to have developed HIV infection by nine months of age were excluded from the study. Additionally, mothers and infants whose samples were used for this study screened negative for potential infection/exposure during pregnancy to malaria (*Plasmodium falciparum)*, Syphilis (*Treponema pallidum*), SARS-COV-2, Dengue (DENV), and Chikungunya (CHKV) using dry red blood spots and sera acquired during pregnancy and at delivery. Screening for congenital Cytomegalovirus (CMV) will also be carried out using neonate urine samples collected during the first two weeks of life.

### Sample collection and processing

All infant specimens collected for this study were obtained from uncomplicated term pregnancies. After delivery, cord blood was collected in citrate phosphate dextrose via puncture of the umbilical vein and was processed within 12 hours of acquisition utilizing a Lymphoprep (STEMCELL Technologies, Vancouver, Canada) gradient. Cord blood mononuclear cells (CBMC) were cryopreserved in freezing medium consisting of 90% fetal bovine serum (FBS, Gemini Bio, Sacramento, CA, USA) and 10% Dimethyl sulfoxide (Millipore Sigma, Burlington, MA) (39). CBMC were stored in vapor phase of liquid nitrogen until shipment to Baltimore, MD, USA for sample processing.

CBMC were thawed, treated with DNAse I (STEMCELL Technologies), and resuspended in complete RPMI 1640 (Thermo-Fisher, Waltham, MA, USA). Cells were stained with anti-CD45 and counted on a Guava easyCyte (Millipore Sigma) and the cell numbers reported as the number of CD45+ events. CBMC were resuspended at 3x10^6^ cells/mL in complete medium and incubated for 6 hours with protein transport inhibitors (Thermo-Fisher, Waltham, MA) or with PMA+ionomycin plus protein transport inhibitors (eBioscience Cell Stimulation Cocktail) (40). After incubation at 37°C, 5% CO_2_ the cells were prepared for analysis.

### Spectral flow cytometry analysis

After a 6-hour incubation, CBMC were stained with Zombie NIR viability dye (Biolegend) for 15 minutes at RT in the dark, followed by a wash. Cells were sequentially stained with cocktails of surface marker mAbs at specific temperatures (**Supplementary Table 1**) with or without tetramers (hMR1 5-OP-RU, hMR1 6-FP, hCD1d PBS-57 and unloaded hCD1d tetramers acquired from the NIH tetramer core) (2, 41, 42) with a wash between steps. The first cocktail of antibodies (primarily against chemokine receptors, **Supplementary Table 1**) was incubated at 37°C for 30 minutes, the second cocktail (including tetramers) at RT for 40 minutes, the third cocktail (against other surface markers) at 4°C for 30 minutes. Following the last incubation, CBMC were treated with BD Pharm Lyse (BD Biosciences) for 3 minutes to lyse remaining red blood cells and immediately washed after the treatment. The cells were then fixed/permeabilized with Cytofix/Cytoperm (BD Biosciences), washed twice, and incubated at RT for 40 minutes with a mix of antibodies specific for intracellular cytokines. After washing, the samples were resuspended in 0.4% PFA and immediately stored at 4°C. All experiments were acquired on a 5 laser Cytek Aurora (Cytek Biosciences, Fremont, CA, USA) the following day, after instrumentation passed automated quality control. The median number of viable lymphocytes that were acquired for fully stained specimens was 6.8 x 10^5^ cells [IQR 5.7 x 10^5^, 8.5 x 10^5^]).

For normalization purposes, each experiment included control PBMC obtained from a single healthy adult donor in a single blood draw. Unmixing controls were pre-optimized following best practices (43–46). The choice of CBMC or PBMC for specific unmixing controls was determined in advance, to ensure that the median fluorescent intensity (MFI) of each single-color fluorophore was greater than or equal to that of the fluorophore in the fully stained sample. The same antibodies were used for the unmixing controls as for the respective fully stained panel. For each experiment, the full set of unstained and single-color control specimens (3-5x10^5^ cells per control) were processed alongside the complete panel specimens. A backup set of single color-unmixing controls using beads (eBiosciences UltraComp Beads Plus, Thermo-Fisher) were processed as the other controls at the start of the experiment series.

Raw .fcs files for unstained and single-color unmixing controls were exported from SpectroFlo (v3.3.0, Cytek Biosciences) to R (R Statistical Software v4.3.1; R Core Team 2023) to evaluate autofluorescence and tandem stability using our R package Luciernaga (47). Unmixing controls exhibiting substantial variation across experimental runs were flagged for scrutiny during the unmixing process and replaced as necessary. Unmixing was carried out in SpectroFlo. The universal negative controls always matched the respective single-color unmixing controls and fully stained specimens in terms of treatment and cell type. The general autofluorescence extract feature was utilized for all samples. We evaluated unmixing by employing 1xN plots in both SpectroFlo and R. When substantial unmixing errors were observed, we substituted brighter single-color unmixing controls from a different experimental run as the main method to resolve unmixing issues.

### Conventional flow cytometry analysis

Cells from every study participant were stained with 4 separate CFC panels, each consisting of 8–9 markers (**Supplementary Table 2**). Following fixable viability staining with Zombie Aqua (Biolegend, San Diego, CA, USA) or Horizon 780 (BD Biosciences, Franklin Lakes, NJ, USA), the CBMC were washed and incubated with a cocktail of surface markers (**Supplementary Table 2**) for 20 minutes at 4°C. After RBC lysis, streptavidin was added for 15 minutes at RT in the dark.

For analysis of intracellular cytokines and cytotoxic effector molecules, the CBMC were fixed/permeabilized using BD Cytofix/Cytoperm (BD Biosciences) and intracellularly stained with an optimized panel of mAbs (**Supplementary Table 2**). Cells were resuspended in 0.4% paraformaldehyde fixation buffer and analyzed within 24 hours on a 4 laser LSR-II flow cytometer (BD Biosciences). The median number of viable lymphocytes that were acquired for fully stained specimens was 4.3 x 10^5^ cells [IQR 3.0 x 10^5^, 5.0 x 10^5^]).

### Cord Blood Vδ2 T lymphocytes function after *in vitro* expansion

To assess Vδ2s cytokine responses and degranulation after expansion, CBMC were cultured in each of the following conditions: A) zoledronic acid monohydrate (ZOL, 0.5 µM, Millipore Sigma) and human recombinant interleukin 2 (IL-2, 20 ng/mL, R&D Systems, Minneapolis, Minnesota, USA); B) Bacillus Calmette-Guerin (BCG, Pasteur strain) at a pre-optimized multiplicity of infection of one (kindly provided by Dr. R. Manganelli, University of Padova) in the presence of IL-2 (20 ng/mL); and C) IL-2 alone as a negative control treatment. The samples were incubated for 17 days at 37°C with 5% CO_2_ and fresh cytokine was added every three days doubling the volume with fresh medium on days 7 and 10. On day 14, only 2 ng/mL of cytokine was added to rest the cells (48). On day 17, the CBMC were counted on a Guava easyCyte (Millipore Sigma) and allocated either to analysis of differentiation/activation post-expansion or restimulation to assess cytokine production and granule mobilization via CFC.

Seventeen days after stimulation, the CBMC were restimulated with anti-γδ TCR (clone B1.1) in 96-well plates coated overnight at 4°C with anti-γδ TCR (Clone B1, diluted 1:100 in PBS, 50 µl/well). The cells were plated in triplicate (2x10^5^ per well) in the presence of anti-CD107a AlexaFluor488 (clone H4A3), GolgiPlug (brefeldin A, 1:1000), and GolgiStop (monensin, 1:1500) (BD Biosciences). Following a 6-hour incubation, the cells were collected and washed with cold PBS, before being processed for CFC as detailed above. At least 1 x 10^5^ lymphocytes were acquired on a LSR II (BD Biosciences) analyzer the following day, as described above.

### Supervised analysis

Following acquisition (for CFC) or unmixing (for SFC), fcs files were imported in FlowJo (v10.10.0, Beckton-Dickinson subsidiary, Ashland, OR, USA). Samples were visualized using the bi-exponential transformation, and a gating template was applied, with manual adjustments to exclude doublets, cell debris, and dead cells. For SFC, events in the live cell gate of each fully stained sample were exported as an individual file to a separate workspace where another gating template for ILT and non-ILT cell subsets was applied and adjusted (**Supplementary Figure 1**). Vδ2 cells were gated as CD7+CD3+Vδ2+ cells, using the clone B6, which is likely to recognize the Vγ9Vδ2 TCR (49). MAITs were gated as either CD7+CD3+CD161^hi^Vα7.2+ or CD7+CD3+CD161^hi^hMR1 5-OP-RU+ cells for anti-TCR- or tetramer-based identification. NKTs were gated as CD7+CD3+Vα24Jα18+ or CD7+CD3+hCD1d PBS-57+ cells for anti-TCR- or tetramer-based identification (2, 41, 42). For CFC, pre-acquisition compensation was generated with single color controls and fine-tuned post-acquisition for individual specimens. A panel-specific gating template was applied and adjusted as needed (**Supplementary Figure 1**). For both methods, data including frequencies, MFI and counts was then exported as .csv files for use in statistical analysis.

### Semi-supervised analysis

Events in the manually gated ILT sub-populations described above were imported from FlowJo to R using the CytoML and flowWorkspace R packages (50, 51). Samples were bi-exponentially transformed, with quality control checks carried out on individual specimens using the PeacoQC and Luciernaga packages (47, 52). Normalization was conducted using either CytoNorm v2.0 or CyCombine packages (53–55), when required based on visual screening for batch effects (due to instrumental variation or other causes). The effect of normalization was visualized by plotting the data before and after normalization for each specimen and cell subset in R. To profile ILT features ex-vivo and explore the heterogeneity of cord blood ILT subsets, we implemented a semi-supervised analytical approach validating unsupervised analysis by manual gating. Using the interactive Shiny App in our R package Coereba (56), manually gated ILT cells from each specimen were visualized as individual bivariate plots. Individual markers were sequentially visualized across all specimens by switching the x-axis parameter while keeping the y-axis parameter constant. Automated minimum-density gating from the openCyto package (57) was used to estimate the split-point between positive and negative events for each given marker and individual. These automated gates were visualized and manually adjusted as needed through Coereba’s RShiny application. The MFI values corresponding to these individualized split-point gates were then exported to a .csv file. The final gate placement for each given marker and individual was validated via their visualization on a 1xN plot using the Luciernaga package.

Following split-point validation, an identity column was generated with Coereba for each cell within an individual specimen, classifying the cells by the expression of their markers. The frequency of positive cells was defined for each marker in every donor by the location of the relative split point. The identity column, alongside metadata corresponding to the individual specimen and treatment, was then appended as new columns in the .fcs file. After this process, all the cells originally identified as specific ILT subsets by manual gates across all individual samples were concatenated in a single .fcs file for each of the two treatments (PMA+ionomycin or unstimulated control). Each concatenated file was imported into FlowJo for dimensionality visualization using the Pairwise Controlled Manifold Approximation (PaCMAP) plugin (58). Markers that had been used to exclude events in the manual gating strategy, or were not expressed after a specific treatment, were not included as parameters to run the PaCMAP. Additionally, we ran other dimensionality visualization methods (tSNE, UMAP and PHATE (59–61)) to compare the output across algorithms.

We manually gated clusters of the resulting PaCMAP on the basis of cell density distribution and marker expression. An individual.fcs file was generated for each cluster within the visualization and brought into R using the CytoML package. Coereba was then used to retrieve and reassemble the stored metadata for individual cells into a “tidy” format (62), allowing calculation of the frequency of cells derived from each individual across the various clusters. Using the stored manually annotated gating information, we calculated median expression frequency for every marker across all individuals in each manually identified cell subset or PaCMAP cluster. Expression levels were summarized as beeswarm plots or heatmaps using the Coereba and gt packages (56, 63). The heatmaps use a combination of colors and symbols to display, for each marker, the median of individual frequencies of positive cells, across ILT clusters. For this study, within the text we defined the analysis of manually gated ILT subsets as “global”, and that of PaCMAP-visualized ILT clusters as “local”. Finally, we brought into the R workspace the manually gated ILT subsets and generated co-expression matrices for the individual markers using the CytoGLMM package (64).

### Statistical analyses

All analyses were performed using R Statistical Software (v4.3.1) (65). For null hypothesis significance testing (NHST), a p-value < 0.05 was considered significant. We assessed normality using D’Agostino Omnibus K2 test (implemented in fBasics R package (66)), visually inspecting the distributions with histogram and Quantile-Quantile plots. Where needed, we compared potential differences in the variance of distributions between exposure groups using the Bartlett’s test. Continuous variables in the text are presented as medians for proportions of positive cells in the parent gate, followed by interquartile ranges (e.g., X% [Y,Z]). Pairwise comparison of median frequencies for two groups were conducted using a two-tailed student’s t-test or a Wilcox non-parametric test for normal or non-normal distributions respectively. For comparisons between three groups, we employed either a One-Way Anova (Type II) or a Kruskal-Wallis test for normal and non-normal distributions, respectively. P-values were adjusted for multiple comparisons using Benjamin Hochberg correction. To assess whether sex-specific differences were independent of HIV/ART exposure status, we fitted two Generalized Linear Models with the stats package using the following formulas: 1) ~ Infant sex + HIV/ART exposure group, where HIV/ART exposure group was a factor with 3 variables (HU, HEU-lo, HEU-hi); and 2) ~ Infant sex + HIV/ART exposure group + Infant sex *HIV/ART exposure.

## Results

### Study population

A total of 75 maternal-fetal pairs were selected (HU = 26, HEU-lo = 24, HEU-hi = 25) among participants enrolled between July 2018 and January 2022, ensuring a balanced estimated gestational age distribution across groups and equal proportions male to female births. Among the mothers with high viral load who initiated ART at the first antenatal care visit (HEU-hi), 80% had undetectable viral load at delivery after a median of 97 days [73, 121] on ART (**Table 1**). Additional demographic information for participants included in this study is detailed in **Table 1**.

**Table 1:** Cohort characteristics.

Demographics	HU N=26*^1^*	HEU-lo N=24*^1^*	HEU-hi N=25*^1^*
Maternal Age (years)	24 (19, 32)	27 (24, 35)	26 (22, 30)
Maternal viral load (screening)	NA (NA, NA)	0 (0, 0)	26,915 (15,732, 93,422)
Detectable viral load (delivery)	0 (NA%)	0 (0%)	5 (20%)
Maternal CD4+ cells/µL (delivery)	NA (NA, NA)	613 (503, 769)	489 (391, 576)
Days between screening and delivery	102 (75, 122)	116 (95, 134)	97 (73, 121)
Infant sex (female)	14 (54%)	12 (50%)	13 (52%)
Estimated Gestational Age, weeks	39.79 (38.14, 40.14)	39.79 (39.07, 40.36)	39.57 (38.71, 40.57)
Parity			
0	7 (27%)	0 (0%)	2 (8.0%)
1	10 (38%)	12 (50%)	11 (44%)
2+	9 (35%)	12 (50%)	12 (48%)

*^1^* Median (Q1, Q3); n (%).

### Frequencies of some Innate-like T cell subsets are altered in HEU infants

**Figure 1:**
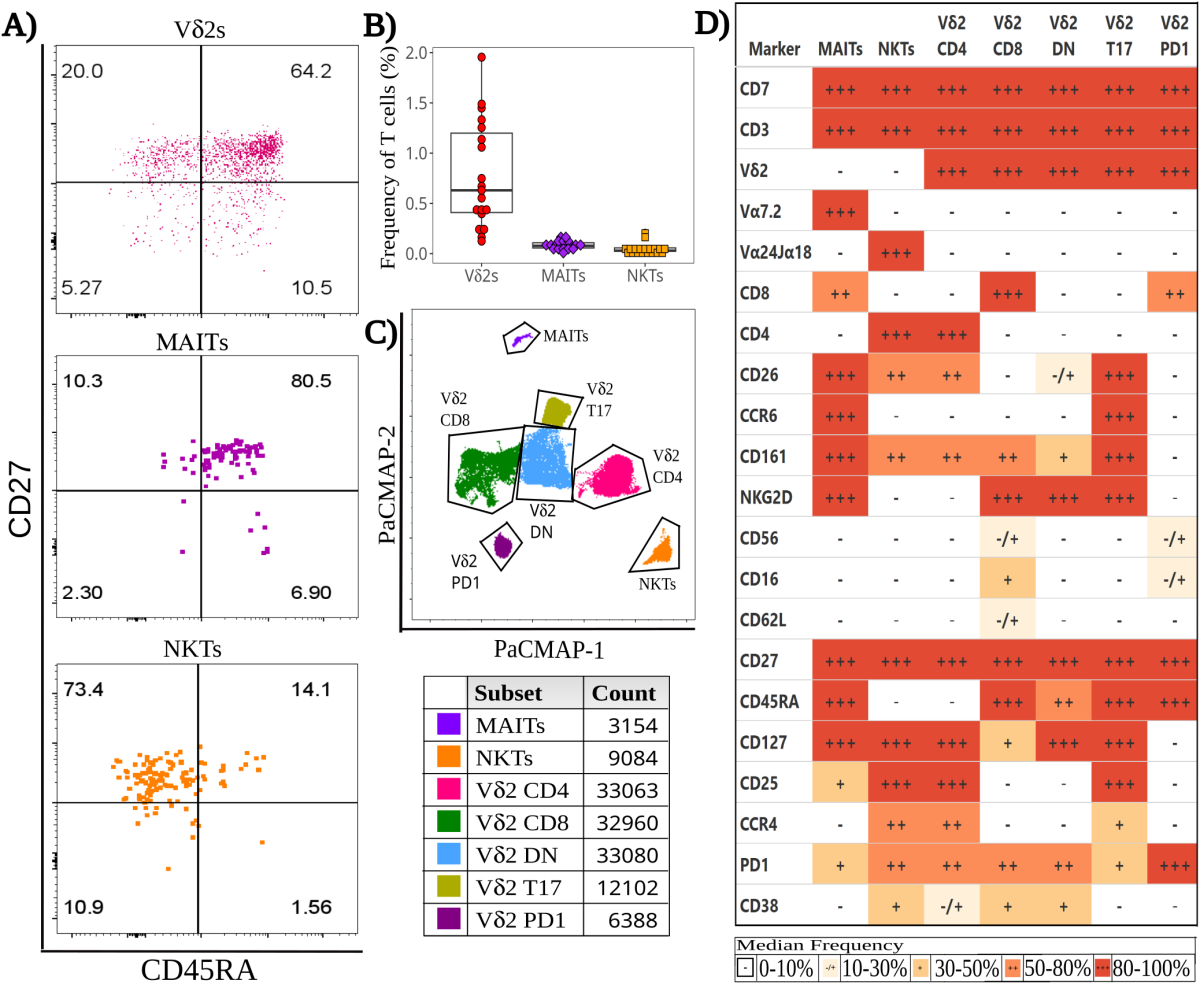
Baseline heterogeneity of cord blood Innate-like T (ILT) cells. Thawed CBMC were stained with a panel of 29 mAbs after a 6-hour incubation at 37°C in the presence of golgi inhibitors. **(A)** Dotplots of a representative specimen obtained from an unexposed (HU) infant, show the expression of CD45RA and CD27 for Vδ2s (top), MAITs (middle), and NKTs (bottom). **(B)** The beeswarm plot displays the frequency of ILT subsets, with individual symbols representing unique HIV unexposed (HU) study participants, boxplots depicting median and IQR, whiskers showing the +/- 1.5 IQR range. **(C)** A PaCMAP dimensionality reduction plot is used to visualize ILT subsets in a concatenated file, including NKT and MAITs as identified by tetramer staining; the gates are based on density-distribution and marker expression, with color code and cell counts included in the legend. **(D)** The heatmap summarizes marker expression for all ILT clusters delineated in **(C)** for all study participants, using a combination of colors and symbols to display, for each marker, the median of individual frequencies of positive cells.

ILT frequencies were determined by applying a manual gating strategy to a detailed 29-color SFC panel (**Supplementary Figure 1**). In neonates born to healthy mothers, Vδ2s were the most abundant ILT subset in T cells (0.693% [0.421, 1.30]) (**Figures 1A, B**), detectable in all CBMC specimens analyzed. MAITs were present at lower frequencies than Vδ2 cells. Of note, the proportion of MAITs identified by tetramer staining (CD3+ CD161^hi^ hMR1 5-OP-RU+ cells, 0.022% [0.017, 0.027]) was lower (p-value: 1x10^-6) than the proportion identified by the antibody specific for the semi-invariant Vα7.2 TCR chain (CD3+ CD161^hi^ Vα7.2+ cells, 0.078% [0.056, 0.108]) (**Supplementary Figure 2A**). NKTs were the least abundant of the three ILT populations (0.053% [0.044, 0.073]) (**Figures 1A, B**). The proportion of NKTs stained with tetramers (CD3+ hCD1d PBS-57+ cells) was not significantly different from the proportion identified by an antibody specific for the semi-invariant Vα24Jα18 (CD3+ Vα24Jα18+ cells; 0.029% [0.023, 0.057]; **Supplementary Figure 2B**).

While the Vδ2 frequency appeared comparable between HU and HEU neonates by SFC (**Supplementary Figure 2C**), an elevated Vδ2 cell frequency was observed in HEU-hi infants by CFC analysis, which included a distinct subset of specimens than the SFC analysis (**Supplementary Figure 2D**). The frequency of MAITs by tetramer staining appeared similar across exposure groups, but it tended to be lower in HEU infants compared to HU infants with anti-TCR Vα7.2, staining (**Supplementary Figure 3A**) and reached a statistically significant difference when HEU-lo and HEU-hi specimens were pooled (**Supplementary Figure 3B**). A similar trend was also noted for NKTs, which were also significantly lower in HEU after pooling the HEU-lo and HEU-hi groups, but only when staining with CD1d tetramers (**Supplementary Figures 3C, D**).

### Innate-like T cell differentiation patterns are similar across HIV/ART exposure groups

To evaluate cell differentiation, we measured the expression of CD45RA and CD27 in ILT subsets (**Figure 1A**, **Supplementary Figure 4A**). CD62L was expressed at low frequencies in ILT, in contrast to adaptive T cell subsets, minimizing its utility for the assessment of differentiation. Most Vδ2 cells were naive (CD45RA+ CD27+, 54.0% [51.0, 70.2]), with a sizable subset of central memory cells (Tcm) (CD45RA- CD27+, 25.1% [16.3, 38.4]) in most individuals (**Supplementary Figure 4A**). Cord blood MAITs were almost all naive (CD27+ CD45RA+, 87.5% [81.8, 92.5]), with only a few neonates displaying any sizable, differentiated subset (**Supplementary Figure 4A**). Results were comparable for cells identified by either anti Vα7.2 or tetramer staining (**Supplementary Figure 4B**). Conversely, NKTs were predominantly Tcm (CD45RA- CD27+, 67.4% [60.3, 77.0]), followed by naive, Tem, and Temra (CD45RA+CD27-) cells (**Supplementary Figure 4A**). NKTs identified by anti-Vα24Jα18+ or tetramers had comparable memory phenotypes (**Supplementary Figure 4C**). The differentiation state of the ILT populations was comparable between HIV/ART exposure groups.

### Semi-supervised analysis of cord blood ILT subsets at baseline highlights Vδ2 cell heterogeneity

To rigorously compare HEU and HU infants, we first employed a semi-supervised analysis to assess the heterogeneity of ILT subsets during homeostasis. Comparing multiple dimensionality reduction and visualization methods (**Supplementary Figure 5**), individual algorithms differed in the extent of splitting or coalescing, but all grouped MAITs and NKTs in a single cluster per subset. Vδ2s clustered by CD4, CD8 and CCR6 expression (**Supplementary Figure 5**), which appears to align with prior studies on Vδ2 cell function (48, 67–70). Comparing the results of the PaCMAP and the heatmap (**Figures 1C, D**), it appears that markers driving cluster formation in the PaCMAP were those expressed at the highest (+++, red) or lowest (-, white) levels by cells within the cluster, aligning with marker co-expression patterns (**Supplementary Figures 6, 7**).

Within the PaCMAP (**Figure 1C**, **Supplementary Figures 5, 6**), cord blood MAITs were primarily CD8+, and most expressed CD161, CD26, CCR6, NKG2D, and CD127 (**Figures 1D**, **Supplementary Figure 8**), while the median proportion of MAITs expressing CD25 or PD1 fell in the 20%-50% range. Of note, <20% of MAITs expressed CD56, CD16, CD62L, CD4 and CCR4 (**Figures 1D**, **Supplementary Figure 8B**). NKTs were primarily CD4+ and uniform in their expression of CD127, with most cells expressing CD25, CCR4 and PD1 (**Figures 1D**, **Supplementary Figure 8C**). The median frequency of expression for CD26 and CD161 was 40%-60%, while CD38 was expressed by 30%-50% of the cells, with the remaining markers expressed by <20% of the cells.

**Figure 2:**
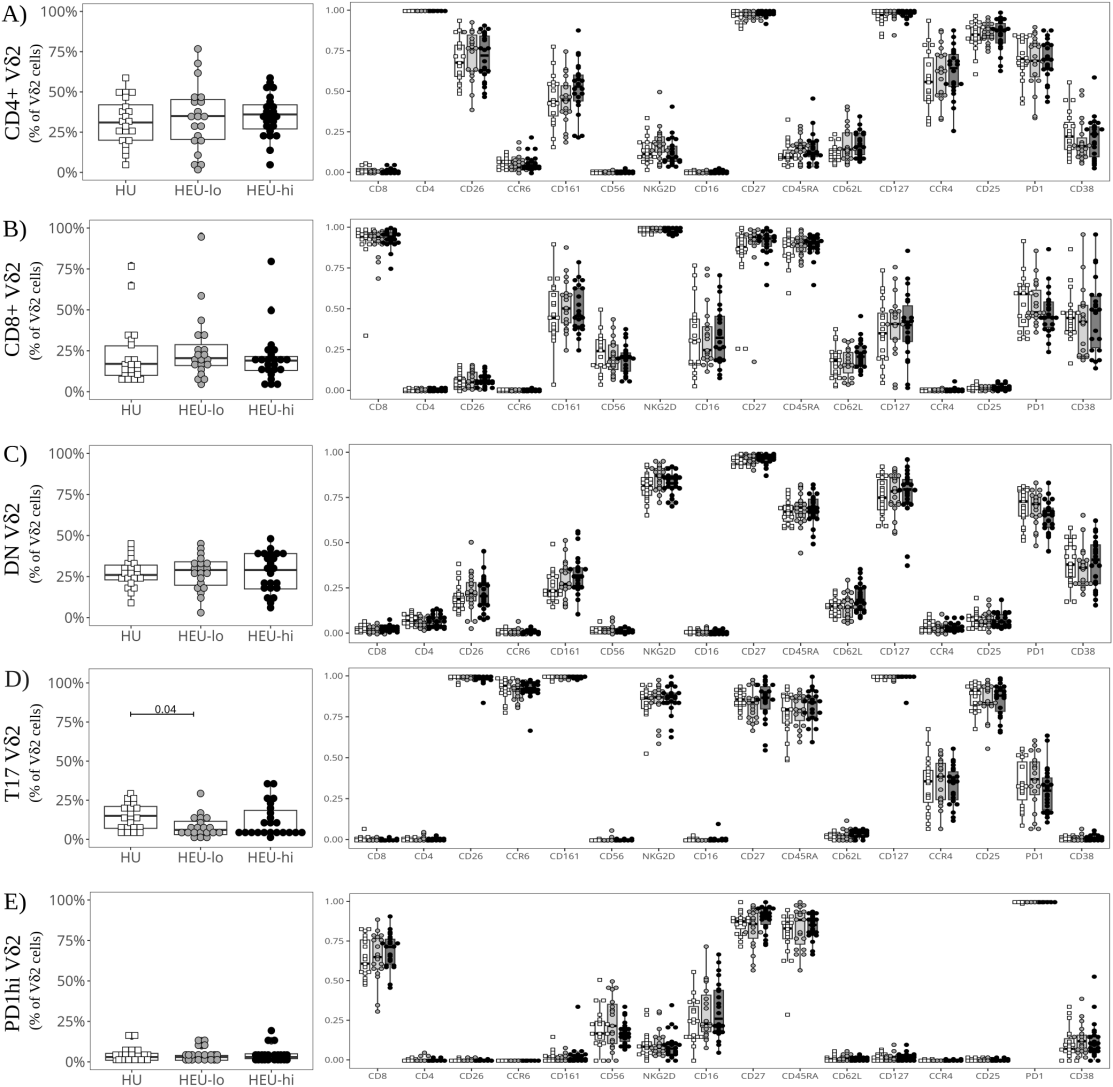
Semi-supervised analysis highlights cord blood Vδ2 cell phenotypic heterogeneity between clusters. The beeswarm plots on the left show the proportion of cells in each Vδ2 PaCMAP cluster (identified in **Figure 1C**) comparing the three exposure groups. The beeswarm plots on the right display the proportion of Vδ2 cells in the respective cluster expressing the individual markers listed on the x-axis. The symbols show individual values, boxplots show median and IQR, with whiskers indicating the +/- 1.5 IQR range. Shape and color identify HIV-exposure status. **(A)** depicts results for the CD4+ PaCMAP cluster, **(B)** for CD8+, **(C)** for DN, **(D)** for T17, and **(E)** for PD1hi.

**Figure 3:**
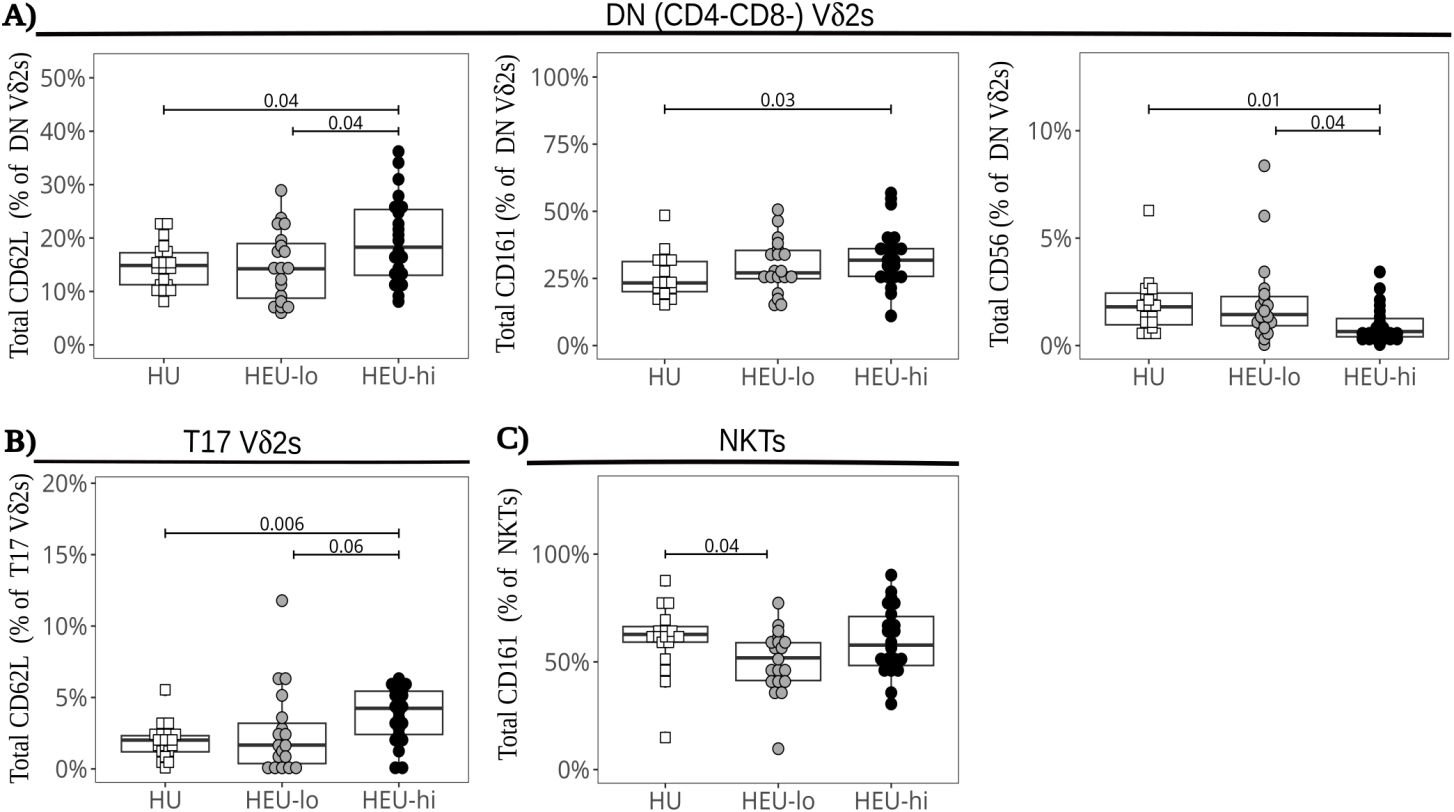
Altered marker expression of cord blood ILT subsets in HEU infants. The beeswarm plots compare the expression of specific markers between exposure groups. Symbols show individual values, boxplots show median and IQR, with whiskers indicating the +/- 1.5 IQR range. Shape and color identify HIV-exposure status. **(A)** Proportions of CD62L+ (left), CD161+ (center), and CD56+ (right) cells in the DN Vδ2 cluster. **(B)** Proportions of CD62L+ cells in the Vδ2 T17 cluster. **(C)** Proportions of CD161+ cells in the NKT PaCMAP cluster.

Vδ2 cells, the most abundant ILT population, were distributed into five clusters. The three most abundant subsets were primarily defined by the presence or absence of CD4 and CD8, the fourth by the expression of CCR6, and the smallest by high levels of PD1 (**Figures 1D**, **2A–E**, **Supplementary Figure 8A**). The CD4+ Vδ2 cluster (35.0% [23.5, 45.0] of total Vδ2s, **Figures 1D**, **2A**), similar in marker expression to NKTs, was predominantly Tcm, with uniform expression of CD127 and most cells positive for CD25. Most cells also expressed CD26, CCR4 (a marker of Th2 polarization) and PD1 (likely akin to a differentiation marker in Vδ2 cells) (40, 69, 70). Approximately 50% of CD4+ Vδ2 cells displayed CD161. NKG2D and CD38 were limited to <20% of the cells, while markers of cytotoxic potential (CD56 and CD16) were barely expressed. The CD8+ Vδ2 cluster (18.5% [12.7, 25.0]) was primarily naïve with uniform expression of NKG2D (**Figures 1D**, **2B**) and intermediate expression of CD161, CD38 and PD1. Minimal to no expression of CD26, CCR6, or CCR4 was noted, suggestive of a Th1-like polarization. A cluster of double-negative (DN; CD4-CD8-, 28.0% [19.7, 35.0]) Vδ2 cells were mostly naive, with Tcm cells being second most represented differentiation subset (29.4%). The majority expressed NKG2D, CD127, and PD1, with intermediate expression of CD26, CD161, CD38 (**Figures 1D**, **2C**). A Vδ2 cluster segregated from the rest of the DN cells, due to its high expression of CCR6, CD26 and CD161, suggestive of Th17 polarization (70). Cells in this cluster, identified as T17 (9.0% [4.0, 16.5], **Figures 1D**, **2D**), were CD4-, CD8-, CD127+ and, despite being naive, most displayed NKG2D and CD25. Less than 50% of the cells expressed PD1. The last Vδ2 cluster was a small subset (present in >85% of individuals, 3.0% [1.5, 5.0]) of mostly naive CD8+ cells characterized by high PD1 (**Figures 1D**, **2E**) and minimal NKG2D expression (similar to the CD4+ cluster). The CD8+ Vδ2 and PD1hi Vδ2 clusters were the only two including a sizable proportion of CD56+ and CD16+ cells (20.1% and 30.2% for the CD8+ and 17.1% and 23.8% for the PD1hi cluster), suggestive of cytotoxic potential, albeit modest. When we compared the exposure groups by using metadata to stratify events within clusters, we observed small differences. HEU-hi neonates, compared to the other two groups, showed significantly higher expression of CD62L in the DN and in the T17 Vδ2 cluster, as well as higher CD161 and lower CD56 in the DN cluster (**Figures 3A, B**). We additionally observed a significantly lower frequency of CD161+ NKT cells in HEU-lo infants compared to the other two groups (**Figure 3C**).

### Analysis of cord blood ILT subsets following stimulation reveals distinct patterns of cytokine production across Vδ2 cell clusters.

**Figure 4:**
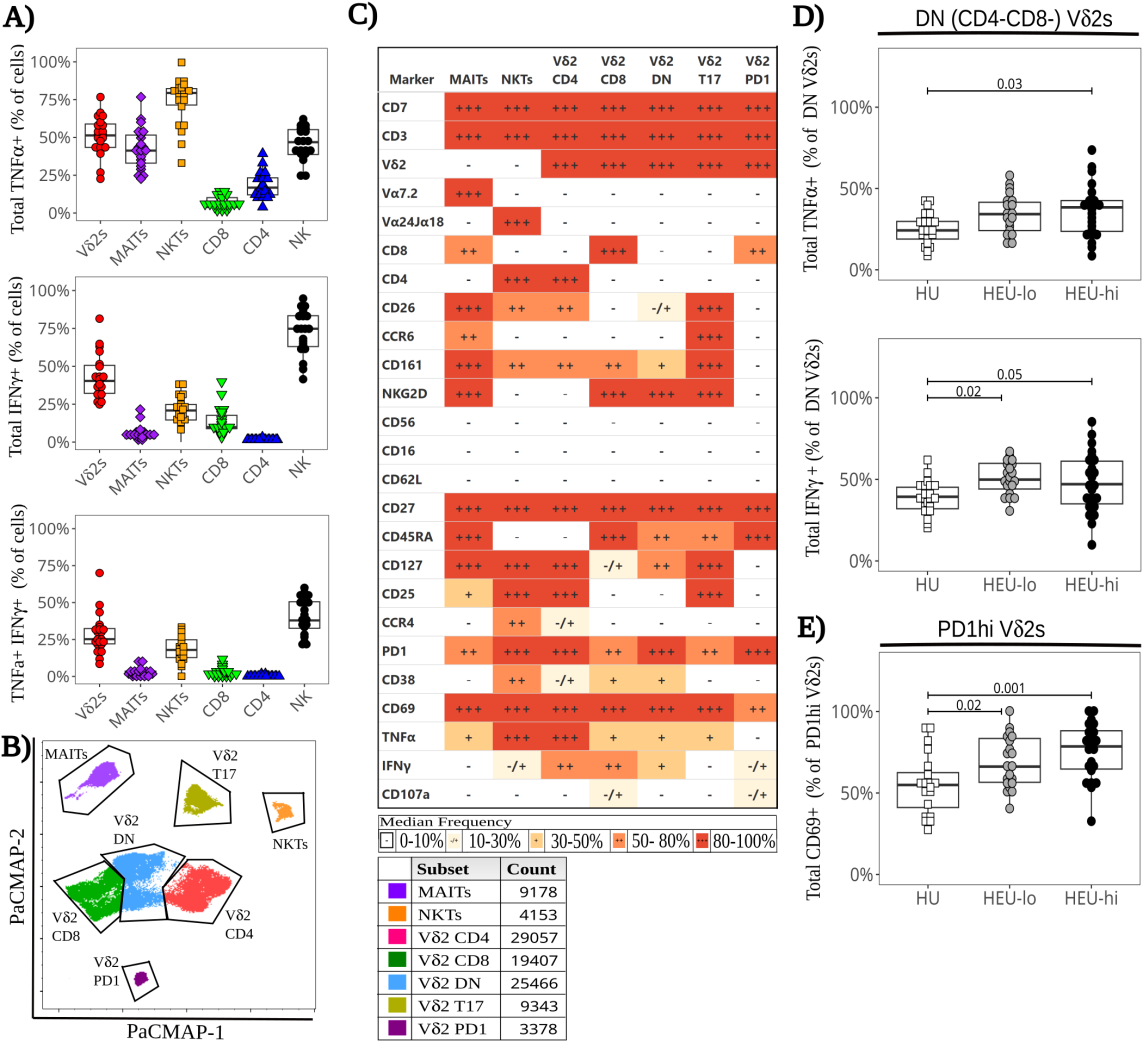
Functional heterogeneity of cord blood ILT in response to polyclonal stimulation ex-vivo with altered cytokine expression in HEU infants. Thawed CBMC were stained with a panel of 29 mAbs after a 6-hour stimulation with PMA plus ionomycin in the presence of golgi inhibitors and CD107a. **(A)** The beeswarm boxplots **c**ompare the frequency of TNFα+ (top), IFNγ+ (middle), and TNFα+ IFNγ+ (bottom) cells for different manually gated cell subsets (listed on the x axis). Symbols depict individual values, boxplots show median and IQR, with whiskers indicating the +/- 1.5 IQR range. Shape and color identify HIV-exposure status. **(B)** A PaCMAP is used to visualize ILT subsets after PMA+ionomycin stimulation, including NKTs and MAITs (identified by anti-TCR staining). The gates are based on density-distribution and marker expression, with color code and cell counts included in the legend. **(C)** The heatmap summarizes marker expression for all ILT clusters delineated in 4B for all study participants, using a combination of colors and symbols to display, for each marker, the median of individual frequencies of positive cells. **(D, E)** The beeswarm plots compare at the local level the expression of specific markers between exposure groups. The symbols show individual values, boxplots show median and IQR, with whiskers indicating the +/- 1.5 IQR range. Shape and color identify HIV-exposure status. **(D)** Proportion of TNFα+ (top) and IFNγ+ (bottom) cells in Vδ2 DN cluster. **(E)** Proportion of CD69+ cells in the Vδ2 PD1hi cluster.

To evaluate ILT function, we assessed their production of cytokines and mobilization of cytotoxic granules in response polyclonal stimulation (**Supplementary Figure 9A**). Comparing the ILT at the global level (as defined in materials and methods), the frequency of TNFα+ cells was highest in NKTs, with a median frequency of 79.4% [71.4, 82.2], while medians ranged between 40% and 55% in the Vδ2 (51.3% [43.4, 58.8]) and MAIT (41.2% [33.0, 51.5]) populations (**Figures 4A**, **Supplementary Figures 9A–C**). The frequency of IFNγ+ cells was lower than TNFα+ cells in all ILT subsets, ranging from a median of 4.3% [2.4, 6.2] in MAITs, to 20.8% [14.5, 24.8] in NKTs, and up to 40.3% [32.1, 50.6] in Vδ2 cells. Polyfunctionality (defined as % TNFα+IFNγ+ cells) was highest in Vδ2s (25.2% [22.0, 32.4]), followed by NKTs (17.9% [12.7, 24.8]) and absent in MAIT cells. Extending the analysis to NK (CD7+CD3-NKG2D+CD161+) and conventional T cells (CD7+CD3+ excluding ILT), the median proportion of TNFα+ cells was 46.8% [38.7, 55.1] for NK cells, 16.7% [12.0, 23.2] for CD4 T cells and 6.4% [3.6, 10.1] for CD8 T cells (**Figure 4A**), while the median proportion of IFNγ+ cells was 74.8% [63.0, 83.3] for NK cells, 9.8% [8.8, 17.6] for CD8 T cells, and 1.1% [0.9, 1.4] for CD4 T cells (**Figure 4A**). Among these three cell populations, only the NK cells had a sizable proportion of polyfunctional cells (37.9% [32.6, 50.5]). Thus, ILT subsets at birth display a cytokine profile more akin to NK cells than conventional T cell subsets.

**Figure 5:**
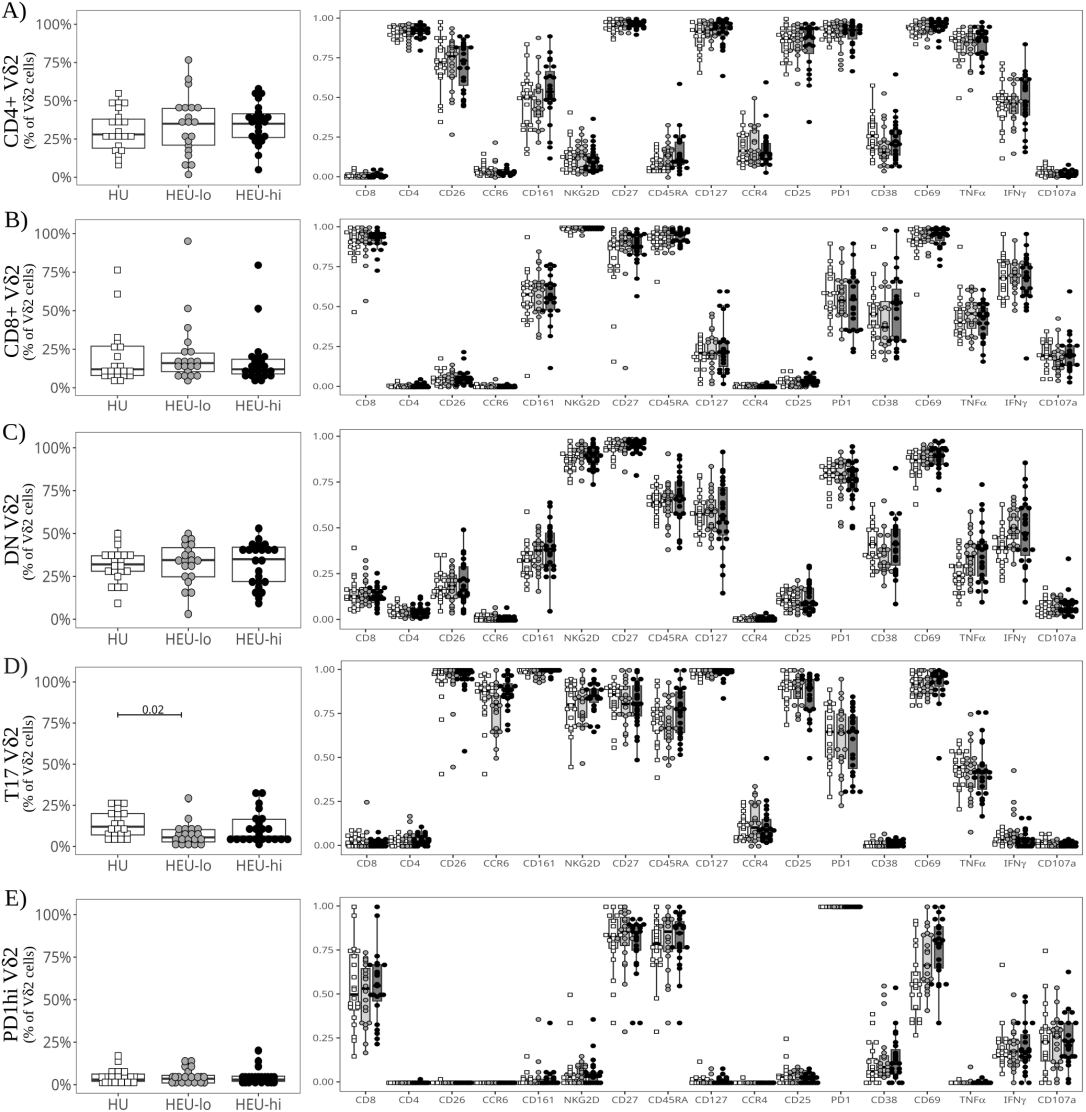
Semi-supervised analysis highlights cord blood Vδ2 T cell functional heterogeneity between clusters after polyclonal stimulation. The beeswarm plots on the left show the proportion of cells in each Vδ2 PaCMAP cluster (from **Figure 4C**), comparing the three exposure groups. The beeswarm plots on the right display the proportion of Vδ2 cells in the respective cluster expressing the individual markers listed on the x-axis. The symbols show individual values, boxplots show median and IQR, with whiskers indicating the +/- 1.5 IQR range. Shape and color identify HIV-exposure status. **(A)** depicts results for the CD4+ PaCMAP cluster, **(B)** for CD8+, **(C)** for DN, **(D)** for T17, and **(E)** for PD1hi.

Dimensionality visualization plots of stimulated cells (**Figure 4B**, **Supplementary Figures 5, 10**) were generated including cytokines and most of the same markers used to generate the PaCMAP for unstimulated cells (**Figure 1C**). The algorithm formed the same clusters identified for unstimulated ILT, with a Vδ2 Th17 cluster segregated from the other Vδ2s (suggestive of distinct features compared to other clusters). MAIT and NKT cells each coalesced in a single cluster, and the results were consistent with the global ILT analysis performed at baseline (**Figures 4A–C**, **Supplementary Figures 9B, C**). The analysis of the Vδ2 clusters at the local level highlighted nuanced cytokine and degranulation profiles across subsets, consistent with our previously published data (48). In the CD4+ Vδ2 cluster, cells uniformly expressed TNFα (86.5% [78.4, 90.4]), with a lower proportion expressing IFNγ (46.2% [38.2, 55.7], **Figures 4C**, **5A**). Less than 4% of the cells expressed CD107a, consistent with a low cytotoxic potential for CD4+ Vδ2 cells (48). Additionally, PD1 was upregulated compared to the unstimulated control (**Figures 2A**, **5A**; p-value 2.4x10^-8^). Cells in the CD8+ Vδ2 cluster primarily expressed IFNγ (68.4% [59.6, 77.3]), with a lower proportion expressing TNFα (42.1% [32.6, 50.2]) (**Figure 4C**, **5B**). A modest frequency of CD107a+ cells was present (18.8% [12.8, 25.1]), consistent with the frequency of CD56+ cells in the control treatment (**Figures 2B**, **5B**), and suggestive of cytotoxic potential. The DN Vδ2 cluster had a cytokine profile comparable to the CD8+ Vδ2 population, but with less mobilization of cytotoxic granules (5.4% [3.4, 9.0]; **Figure 4C**, **5C**). Stimulation induced a decrease in CD127 expression (**Figures 2C**, **5C**; p-value 2.0x10^-6^). Cells in the T17 Vδ2 cluster produced intermediate levels of TNFα (42.4% [34.2, 48.9]), but no IFNγ, and did not mobilize cytotoxic granules (**Figure 4C**, **5D**). The frequency of PD1+ cells was elevated after stimulation (**Figures 2D**, **5D**; p-value 6.0x10^-7^). This functional profile paralleled the activation pattern observed for MAITs. The PD1hi Vδ2 cluster had lower levels of CD69 expression (65.7% [54.6, 82.2]) compared to the other Vδ2 clusters (which were uniformly positive for this activation marker) and displayed a distinct cytokine profile: <1% of the cells produced TNFα, only 16.6% [11.6, 23.7] produced IFNγ and 22.2% [12.8, 32.4] were CD107a+ (**Figures 4C**, **5E**), indicative of modest cytotoxic potential, consistent with the levels of CD56 and CD16 expression observed for unstimulated PD1hi cells (**Figure 2E**, **5E**).

When comparing exposure groups at the ILT global level (as described in materials and methods), we observed no significant difference in cytokine production for any ILT population (**Supplementary Figure 9**). HEU-hi neonates, however, displayed substantial heterogeneity between specimens in the frequency of IFNγ+ Vδ2 cells, with a significantly wider range of positivity (20%-80% of IFNγ+ cells) compared to the other two groups (30%-60% among HEU-lo and HU, p-value 0.03). While CCR6 was downmodulated after stimulation in all exposure groups, the proportion of CCR6+ Vδ2 cells was significantly lower in HEU-lo infants compared to HU infants (**Supplementary Figure 11**). We observed no significant differences across exposure groups in the frequency of individual clusters (**Figures 5A–E**), with the exception of the T17 Vδ2 population, which was lower in HEU-lo infants after stimulation (**Figure 5D**, p-value 0.02), consistent with a CCR6 down-modulation. However, a more granular comparison of PaCMAP Vδ2 clusters between exposure groups revealed differential upregulation of the activation marker CD69 in response to stimulation and differences in cytokine responses. Importantly, the DN Vδ2 cluster displayed a significantly higher proportion of TNFα+ and IFNγ+ in both HEU groups compared to the HU neonates (**Figure 4D**), consistent with higher frequencies of CD69+ cells (p-value 0.05 for both HU vs. HEU-lo and HU vs. HEU-hi). For the PD1hi Vδ2 cluster, the proportion of CD69+ cells was higher in both HEU groups compared to the HU infants, possibly suggestive of a lower activation threshold in the HEU neonatal clusters (**Figure 4E**).

### Vδ2 cell responses after *in vitro* expansion

**Figure 6:**
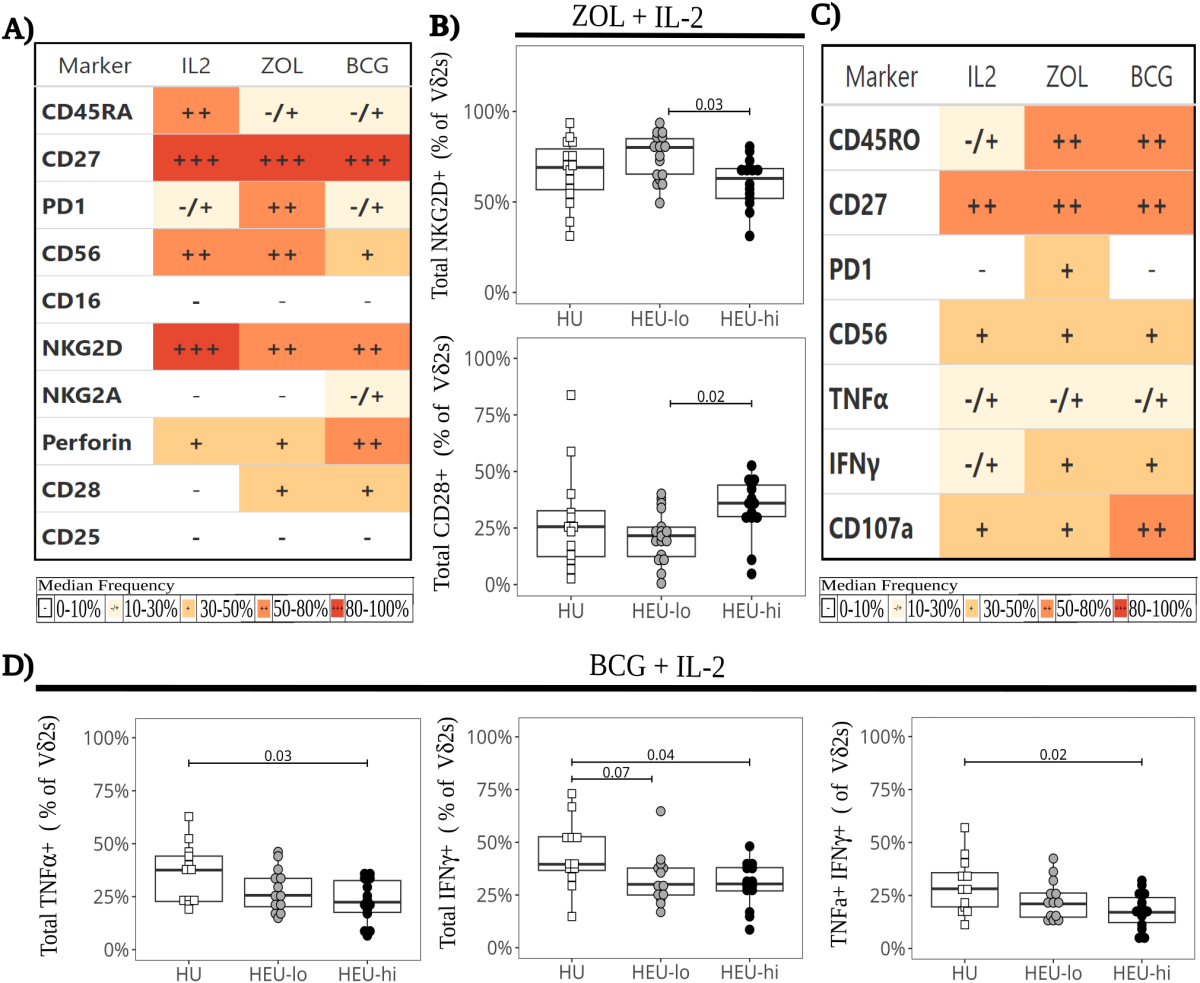
Lower cytokine production in BCG-expanded Vδ2 cells following restimulation in HEU infants. Vδ2 cells were stimulated *in vitro* with ZOL+IL-2, BCG+IL-2, or IL-2 alone and expanded in culture for 17 days. Expanded Vδ2 cells were restimulated with plate-bound anti-γδ TCR antibody **(A)** The heatmap summarizes marker expression of Vδ2 cells by culture condition on day 17 (before restimulation), using a combination of colors and symbols to display, for each marker, the median of individual frequencies of positive cells. **(B)** The beeswarm plots compare the expression of specific markers between exposure groups 17 days after ZOL stimulation (before restimulation), showing individual values, median and IQR, with whiskers showing the +/- 1.5 IQR range for NKG2D+ (left) and CD28+ (right) Vδ2 cells. **(C)** The heatmap summarizes by culture condition the Vδ2 cell cytokine production and degranulation in response to restimulation, using a combination of colors and symbols to display, for each marker, the median of individual frequencies of positive cells. **(D)** The beeswarm plots compare exposure groups for cytokine production by BCG-expanded Vδ2 T cells. The response to restimulation is displayed as the frequency of TNFα+ (left), IFNγ+ (middle), and polyfunctional TNFα+ IFNγ+ (right) Vδ2 cells. The symbols show individual values, boxplots show median and IQR, with whiskers indicating the +/- 1.5 IQR range. Shape and color identify HIV-exposure status.

After expansion with BCG or ZOL (**Supplementary Figure 12**, **Supplementary Table 2**), the frequency of naïve Vδ2 cells declined (median 54.0% *ex vivo*, 15.4% BCG, 10.9% ZOL; p-value 7x10^12^ and 1.3x10^12^ for BCG and ZOL respectively) as they differentiated into Tcm (median 25.1% *ex vivo*, 63.4% BCG, 70.0% ZOL; p-value 7.1x10^7^ and 7.1x10^7^ for BCG and ZOL respectively; **Supplementary Figures 4, 13**). Expansion with either ZOL and BCG led to a lower proportion of NKG2D+ cells and a higher proportion of CD28+ cells compared to the IL2 control (**Figure 6A**, **Supplementary Figure 13**). ZOL stimulation also resulted in a higher frequency of PD1+ cells compared to BCG and IL2, consistent with our previously published data (40, 48) (**Figure 6A**, **Supplementary Figure 13**). We did not observe any statistically significant differences in Vδ2 cell frequencies between exposure groups following expansion (data not shown). In terms of marker expression, after ZOL expansion, HEU-hi infants displayed higher proportions of CD28+ Vδ2 cells compared to HEU-lo infants (**Figure 6B**), while HEU-lo infants had higher NKG2D levels compared to the HEU-hi infants (**Figure 6B**). This suggests that distinct underlying functional potential arising from differentiation may be present between the HEU groups (71). However, we did not observe statistically significant differences in perforin expression between exposure groups for any culture condition. We next re-stimulated expanded Vδ2 cells with plate-bound anti-γδTCR to assess IFNγ and TNFα production as well as degranulation (surface CD107a expression, **Figure 6C**). We did not observe statistically significant differences between exposure groups for the ZOL or IL-2 expanded cells (**Figure 6C**, **Supplementary Figure 14**). However, following culture with BCG + IL-2, HEU-hi infants displayed lower frequencies of TNFα+, IFNγ+, and polyfunctional TNFα+IFNγ+ cells compared to HU infants (**Figure 6D**).

### Sex-differences in cord blood ILT subsets

**Figure 7:**
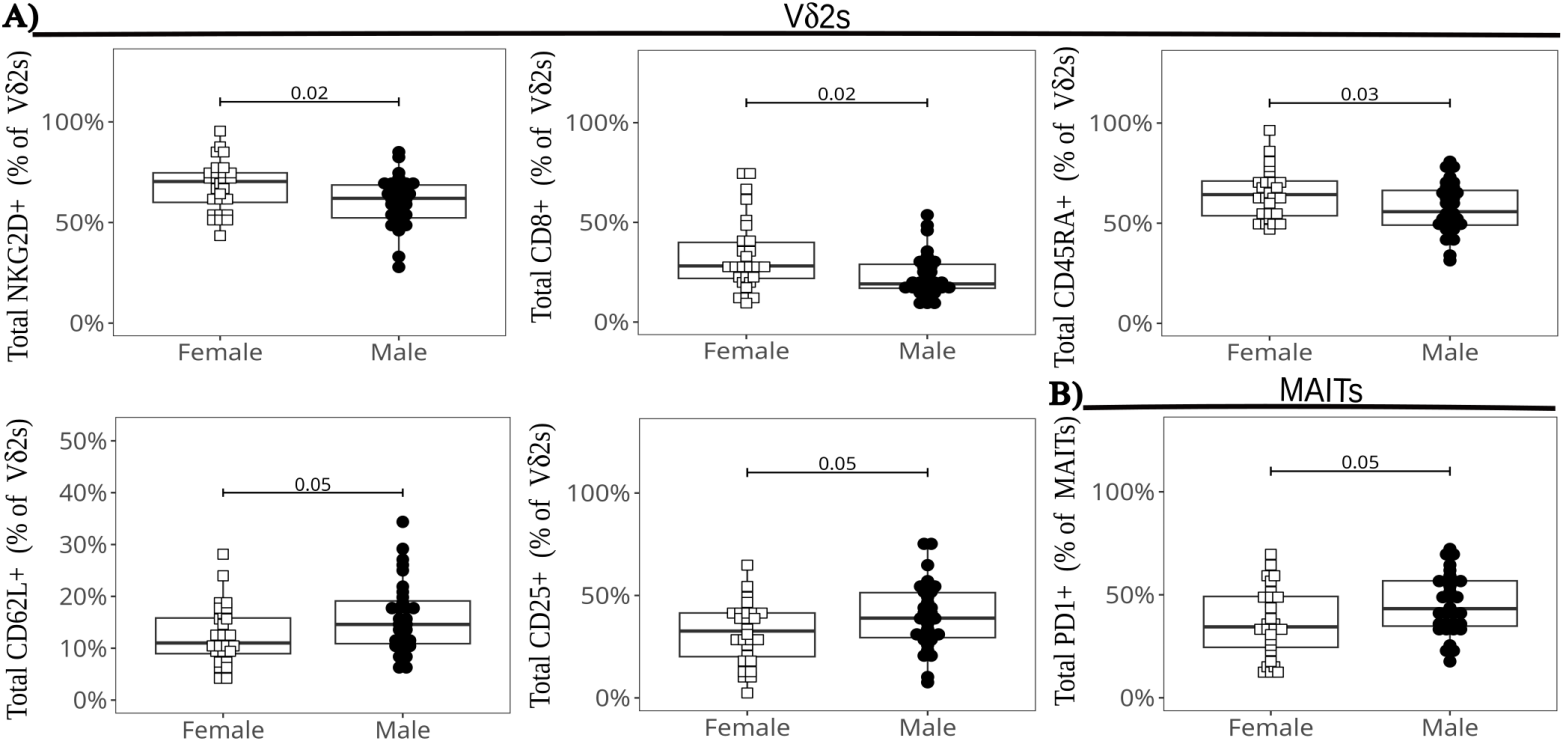
Differences in ILT global marker expression by infant sex. The beeswarm plots show the differentially expressed markers in Vδ2 cells **(A)**, and in MAIT cells **(B)**. The symbols show individual values and the boxplots show median and IQR, with whiskers indicating the +/- 1.5 IQR range. Shape and color identify infant sex.

We compared ILT frequencies and marker expression at baseline by infant sex, pooling all infants regardless of HIV exposure groups (given the lack of major differences between exposure groups at the global level) (**Figure 7**). We observed the following differences in female compared to male neonates: significantly higher frequencies of CD8+, NKG2D+, and CD45RA+ cells and lower proportions of CD62L+ and CD25+ cells in the Vδ2 cell subset (**Figure 7A**); and significantly lower frequencies of PD-1+ MAIT cells (**Figure 7B**). These markers (except CD62L and PD1) were still differentially expressed after PMA+ionomycin stimulation (data not shown). To confirm our observations, we employed a generalized-linear model comparison to account for the contribution of HIV/ART exposure. The results remained statistically significant for sex for all the above markers, with CD62L in Vδ2 cells also exhibiting an interaction with HIV/ART exposure for the HEU-hi group (**Supplementary Table 3**). Of note, a there were no differences between males and females in the production of cytokines or cytotoxic granule release after stimulation both ex-vivo or post-expansion (**Supplementary Figure 15**).

## Discussion

This study demonstrates that the use of SFC for samples of limited cell quantity, such as human pediatric specimens, allows in-depth examination of low frequency subsets, including ILT, that neither mass cytometry nor single cell RNA seq approaches could analyze in a cost-effective manner without prior enrichment. Consistent with our previous findings, we observed extensive heterogeneity in Vδ2 cells, in contrast to the more homogeneous characteristics of NKT and MAIT cells.

Despite a heightened pro-inflammatory milieu reported at the fetal-maternal interface among pregnant women living with HIV, we only observed modest differences at the global ILT level between HU and HEU infants. The differences were represented primarily by altered MAIT and NKT frequencies for pooled HEU infant groups and higher proportions of cells expressing specific trafficking or activation markers. At the local Vδ2 level, the large cluster of DN cells displayed significantly higher proportions of IFNγ+, TNFα+ and polyfunctional cells in HEU-hi compared to HU and HEU-lo neonates. However, after culture with BCG, the HEU-hi group displayed lower proportions of TNFα+ and IFNγ+ Vδ2 cells compared to HU neonates in response to TCR-mediated restimulation, suggesting a possible defect in recall responses. ZOL, an inhibitor of the enzyme FPP synthase, indirectly and potently stimulates Vδ2 cells by inducing upregulation of IPP, the endogenous intermediate of cholesterol biosynthesis sensed by Vδ2 cells (72–74). BCG directly stimulates Vδ2 cells via the production of HMBPP, an intermediate of non-mevalonate pathway of cholesterol biosynthesis, but is less potent that ZOL for cord blood Vδ2 cells stimulation (75–78). Since no differences in response to restimulation were evident for cells expanded with the potent zoledronate, the defect is likely subtle and only measurable after mild stimulation. HEU-hi infants also displayed significantly wider range of IFNγ+ Vδ2 cells following *ex vivo* polyclonal stimulation compared to the other two groups, which was not accounted for by the Vδ2 cluster composition. The heterogeneity in maternal cumulative viremia (79) and time of ART initiation during pregnancy likely resulted in disparate HIV/ART exposure among HEU-hi neonates, which may have contributed to our observation.

Our data suggest that ILT cells are mildly, if at all, perturbed in neonates born to women with HIV who started ART before conception. However, modest differences in cytokine production (*ex vivo* or after culture) and in a few phenotypic markers, noted for HEU-hi neonates, suggest that immune perturbation may be more marked in this exposure group. The results are in agreement with prior studies that showed small or no differences between HU and HEU infants in terms of immune responses and/or clinical outcomes when the mothers are on ART (25, 29). In particular, a study conducted in Belgium showed no difference in the risk of hospitalization between HU and HEU infants born to women that started ART before pregnancy, but a significantly higher risk for HEU infants born to women that initiated ART during pregnancy, correlating with heightened cord blood monocyte activation (25). Therefore, ART initiation before pregnancy may be beneficial not only for the mother, but also for the infant. Initiation of ART preconception may mitigate fetal immune perturbation by limiting exposure to high maternal viremia and/or inflammation. However, the timing of initiation could also prevent unintended effects on the developing fetal immune system that may occur when ART is started during pregnancy. The current study design does not allow us to discriminate between these possibilities.

We characterized the phenotype and functional profiles of cord blood ILT at baseline and following polyclonal stimulation. We observed extensive heterogeneity in Vδ2 cells based on the expression of surface markers and patterns of cytokine responses, consistent with prior single-cell RNA seq data published for a small number of samples (70). While both NKT and MAIT cells appeared more homogeneous, the low abundance of these two subsets may hinder our ability to identify and measure minor NKT and MAIT clusters. CITE-seq analyses of pre-enriched subsets could validate and expand our current findings. In contrast with a previous study, we observed no significant differences in the marker expression of cord blood MAITs or NKTs defined by tetramer or by their corresponding anti-TCR antibody staining (41, 42, 80). The tetramers performed consistently based on internal normalization and unloaded controls. The apparent discrepancy between our results and those of Swarbrick et al. is most likely the result of differences in experimental set-up and gating strategy (6, 81).

The Vδ2 cell phenotypic heterogeneity appeared to correspond, at least in part, to functional characteristics, with different Vδ2 clusters mediating distinct cytokine responses. The three main clusters of Vδ2 cells identified by PaCMAP (CD4+, CD8+, DN) were skewed towards a Th1 response, producing both IFNγ and TNFα at varying levels. The T17 cluster, characterized by expression of CCR6 and CD26, produced TNFα but no IFNγ, while the PD1hi subset expressed very low levels of IFNγ, with no TNFα production. Of note, the CD4+ cluster, which includes the most differentiated Vδ2 cells based on surface markers, shared phenotypic (CCR4, CD25, and CD26) and functional features with NKT cells, with lymphocytes in both clusters secreting more TNFα than IFNγ. Conversely, T17 cells share phenotypic properties with MAIT cells (CD161, CCR6, CD26) and neither subset appears to produce IFNγ in any of the donors analyzed.

Throughout the study we noted small but consistent differences between male and female neonates in the expression of surface markers (CD62L, PD1, NKG2D, CD45RA and CD8) across multiple subsets, predominantly Vδ2 cells. ILT subset frequencies did not account for the observed observed differences. These disparities remained significant after controlling for other variables, such as exposure and gestational age. Preliminary analyses of other cell subsets (NKs and conventional αβ T cells) confirmed differential expression of the same markers between male and female neonates. Importantly, the sex-related differences did not appear to have functional implications, since cytokines, CD69, and CD107a expression were comparable between males and females. Previous studies have reported differences in inflammatory cytokines, androgen receptors, and FoxP3+ Tregs during pregnancy with male versus female neonates (82). Whether our observation is generalizable, or unique to our system, remains to be determined.

Several study limitations should be considered when interpreting our results. While we have maternal clinical and HIV screening information at enrollment and delivery (as well as screening for other common pathogens), we cannot exclude that some women contracted undiagnosed or subclinical infections during pregnancy. Our sample size was relatively small, and, given the observed heterogeneity for multiple variables, we were likely underpowered to detect small but biologically relevant differences across exposure groups. All the specimens were cryopreserved and thawed, which may reduce cell viability and affect marker expression. Our observations for ILT, that respond to microbial metabolites, may be impacted by dietary/microbiota, environmental and geographic factors. Additionally, there may be temporal variability, and we assessed infant responses only at a single time point (at birth). These limitations, however, do not diminish the robustness of our analytical approach towards baseline neonatal ILT physiology, which allowed us to rigorously assess these rare cell subsets and characterize them in neonates born to women living with HIV. While the first full spectrum analyzer became commercially available in 2013 (83), the implementation of this approach has become significantly more widespread in the last 5 years. The current trend is to repurpose packages and data analysis tools originally developed for conventional/mass cytometry or single cell RNA-seq for use in SFC. This practice, however, may not fully leverage SFC’s core strengths, which include increased breadth compared to CFC and enhanced enhanced time/cost effectiveness compared to mass cytometry (often enabling improved depth). In addition, repurposing does not appropriately mitigate the variation in unmixing (due to autofluorescence, signature variance of unmixing controls, and tandem fluorophore degradation), which is unique to spectral cytometry. For example, data analysis tools initially intended for mass cytometry rarely handle the number of events acquired in SFC, making downsampling an obligatory step to use those tools. This approach does not fully leverage the depth of information obtained with SFC, potentially limiting our understanding of the immune system heterogeneity. Building tools capable of handling the unique SFC features and scalable to increasingly complex panels would enable a smoother integration into existing bioinformatic workflows for downstream analysis.

To help characterize the factors contributing to unmixing variance, we created an R package called Coereba, which enables the retention of metadata and manually annotated gating information for individual cells through subsequent unsupervised analytical approaches. Given this feature, Coereba facilitates data exploration, including testing and validation of unsupervised algorithms for quality control and normalization.

In conclusion, we leveraged SFC to characterize rare subsets in neonates and optimized a panel (as well as analysis tools and code) that provides a backbone for ILT analysis and can be built upon as new fluorophore and additional conjugated antibodies become available. Our results, obtained comparing HIV-unexposed and two groups of HEU infants, suggest that ILT are mildly, if at all, perturbed in HEU-lo, born to women who started ART before conception, but showed that the Vδ2 cell subset displays modest differences in HEU-hi neonates, born to women who started ART during pregnancy. This data, in alignment with prior literature, is consistent with the hypothesis that starting women on ART before conception is an effective approach to mitigating infant immune perturbation.

## Data Availability

The .fcs files generated for this study are available via the ImmPort repository (study accession SDY3080). The R code used for the analyses and our R packages are available on GitHub (DavidRach/CordBloodILT; DavidRach/Coereba; DavidRach/Luciernaga). Additional data and code availability details can be found in the **Supplementary Material**.

## References

[1] Awad W Ler GJM Xu W Keller AN Mak JYW Lim XY. The molecular basis underpinning the potency and specificity of mait cell antigens. Nat Immunol. (2020) 21:400–11. doi: 10.1038/s41590-020-0616-6, PMID: 32123373

[2] Eckle SB Birkinshaw RW Kostenko L Corbett AJ McWilliam HE Reantragoon R. A molecular basis underpinning the T cell receptor heterogeneity of mucosal-associated invariant T cells. J Exp Med. (2014) 211:1585–600. doi: 10.1084/jem.20140484, PMID: 25049336 PMC4113946

[3] Melandri D Zlatareva I Chaleil RAG Dart RJ Chancellor A Nussbaumer O. The gammadeltatcr combines innate immunity with adaptive immunity by utilizing spatially distinct regions for agonist selection and antigen responsiveness. Nat Immunol. (2018) 19:1352–65. doi: 10.1038/s41590-018-0253-5, PMID: 30420626 PMC6874498

[4] Godfrey DI Uldrich AP McCluskey J Rossjohn J Moody DB. The burgeoning family of unconventional T cells. Nat Immunol. (2015) 16:1114–23. doi: 10.1038/ni.3298, PMID: 26482978

[5] Mayassi T Barreiro LB Rossjohn J Jabri B. A multilayered immune system through the lens of unconventional T cells. Nature. (2021) 595:501–10. doi: 10.1038/s41586-021-03578-0, PMID: 34290426 PMC8514118

[6] Ben Youssef G Tourret M Salou M Ghazarian L Houdouin V Mondot S. Ontogeny of human mucosal-associated invariant T cells and related T cell subsets. J Exp Med. (2018) 215:459–79. doi: 10.1084/jem.20171739, PMID: 29339446 PMC5789419

[7] Moreira-Teixeira L Resende M Coffre M Devergne O Herbeuval JP Hermine O. Proinflammatory environment dictates the il-17-producing capacity of human invariant nkt cells. J Immunol. (2011) 186:5758–65. doi: 10.4049/jimmunol.1003043, PMID: 21478400

[8] Papadopoulou M Dimova T Shey M Briel L Veldtsman H Khomba N. Fetal public vgamma9vdelta2 T cells expand and gain potent cytotoxic functions early after birth. Proc Natl Acad Sci U.S.A. (2020) 117:18638–48. doi: 10.1073/pnas.1922595117, PMID: 32665435 PMC7414170

[9] Raffetseder J Lindau R van der Veen S Berg G Larsson M Ernerudh J. Mait cells balance the requirements for immune tolerance and anti-microbial defense during pregnancy. Front Immunol. (2021) 12:718168. doi: 10.3389/fimmu.2021.718168, PMID: 34497611 PMC8420809

[10] Solders M Gorchs L Erkers T Lundell A-C Nava S Gidlöf S. Mait cells accumulate in placental intervillous space and display a highly cytotoxic phenotype upon bacterial stimulation. Sci Rep. (2017) 7:6123. doi: 10.1038/s41598-017-06430-6, PMID: 28733576 PMC5522401

[11] Solders M Gorchs L Tiblad E Gidlöf S Leeansyah E Dias J. Recruitment of mait cells to the intervillous space of the placenta by placenta-derived chemokines. Front Immunol. (2019) 10:1300. doi: 10.3389/fimmu.2019.01300, PMID: 31244846 PMC6563723

[12] Anderson J Thang CM Thanh LQ Dai VTT Phan VT Nhu BTH. Immune profiling of cord blood from preterm and term infants reveals distinct differences in pro-inflammatory responses. Front Immunol. (2021) 12:777927. doi: 10.3389/fimmu.2021.777927, PMID: 34790206 PMC8591285

[13] Odorizzi PM Feeney ME. Impact of in utero exposure to malaria on fetal T cell immunity. Trends Mol Med. (2016) 22:877–88. doi: 10.1016/j.molmed.2016.08.005, PMID: 27614925 PMC5048621

[14] Lim AI McFadden T Link VM Han SJ Karlsson RM Stacy A. Prenatal maternal infection promotes tissue-specific immunity and inflammation in offspring. Science. (2021) 373:6558. doi: 10.1126/science.abf3002, PMID: 34446580

[15] Babik JM Cohan D Monto A Hartigan-O’Connor DJ McCune JM. The human fetal immune response to hepatitis C virus exposure in utero. J Infect Dis. (2011) 203:196–206. doi: 10.1093/infdis/jiq044, PMID: 21288819 PMC3071071

[16] Garcia-Flores V Romero R Xu Y Theis KR Arenas-Hernandez M Miller D. Maternal-fetal immune responses in pregnant women infected with sars-cov-2. Nat Commun. (2022) 13:320. doi: 10.1038/s41467-021-27745-z, PMID: 35042863 PMC8766450

[17] Waasdorp Hurtado C Golden-Mason L Brocato M Krull M Narkewicz MR Rosen HR. Innate immune function in placenta and cord blood of hepatitis C – seropositive mother-infant dyads. PloS One. (2010) 5:e12232. doi: 10.1371/journal.pone.0012232, PMID: 20814429 PMC2923602

[18] Le Roux SM Abrams EJ Donald KA Brittain K Phillips TK Zerbe A. Infectious morbidity of breastfed, hiv-exposed uninfected infants under conditions of universal antiretroviral therapy in South Africa: A prospective cohort study. Lancet Child Adolesc Health. (2020) 4:220–31. doi: 10.1016/s2352-4642(19)30375-x, PMID: 31932246 PMC7235356

[19] Slogrove AL Goetghebuer T Cotton MF Singer J Bettinger JA. Pattern of infectious morbidity in hiv-exposed uninfected infants and children. Front Immunol. (2016) 7:164. doi: 10.3389/fimmu.2016.00164, PMID: 27199989 PMC4858536

[20] Abu-Raya B Kollmann TR Marchant A MacGillivray DM. The immune system of hiv-exposed uninfected infants. Front Immunol. (2016) 7:383. doi: 10.3389/fimmu.2016.00383, PMID: 27733852 PMC5039172

[21] Amenyogbe N Dimitriu P Cho P Ruck C Fortuno ES 3rd Cai B. Innate immune responses and gut microbiomes distinguish hiv-exposed from hiv-unexposed children in a population-specific manner. J Immunol. (2020) 205:2618–28. doi: 10.4049/jimmunol.2000040, PMID: 33067377 PMC7653510

[22] Dirajlal-Fargo S Mussi-Pinhata MM Weinberg A Yu Q Cohen R Harris DR. Hiv-exposed-uninfected infants have increased inflammation and monocyte activation. AIDS. (2019) 33:845–53. doi: 10.1097/qad.0000000000002128, PMID: 30649056 PMC6494115

[23] Dzanibe S Lennard K Kiravu A Seabrook MSS Alinde B Holmes SP. Stereotypic expansion of T regulatory and th17 cells during infancy is disrupted by hiv exposure and gut epithelial damage. J Immunol. (2022) 208:27–37. doi: 10.4049/jimmunol.2100503, PMID: 34819390 PMC8702481

[24] Garcia-Knight MA Nduati E Hassan AS Gambo F Odera D Etyang TJ. Altered memory T-cell responses to bacillus calmette-guerin and tetanus toxoid vaccination and altered cytokine responses to polyclonal stimulation in hiv-exposed uninfected Kenyan infants. PloS One. (2015) 10:e0143043. doi: 10.1371/journal.pone.0143043, PMID: 26569505 PMC4646342

[25] Goetghebuer T Smolen KK Adler C Das J McBride T Smits G. Initiation of antiretroviral therapy before pregnancy reduces the risk of infection-related hospitalization in human immunodeficiency virus-exposed uninfected infants born in a high-income country. Clin Infect Dis. (2019) 68:1193–203. doi: 10.1093/cid/ciy673, PMID: 30215689 PMC13375565

[26] Jones CI Rose SL Shutt A Cairo C Bourgeois NM Charurat M. Maternal hiv status skews transcriptomic response in infant cord blood monocytes exposed to bacillus calmette–guerín. AIDS. (2021) 35:23–32. doi: 10.1097/qad.0000000000002706, PMID: 33048873 PMC7718394

[27] Mazzola TN Da Silva MTN Abramczuk BM Moreno YMF Lima SCBS Zorzeto TQ. Impaired bacillus calmette–guérin cellular immune response in hiv-exposed, uninfected infants. AIDS. (2011) 25:2079–87. doi: 10.1097/qad.0b013e32834bba0a, PMID: 21866040

[28] Miles DJ Gadama L Gumbi A Nyalo F Makanani B Heyderman RS. Human immunodeficiency virus (Hiv) infection during pregnancy induces cd4 T-cell differentiation and modulates responses to bacille calmette-guerin (Bcg) vaccine in hiv-uninfected infants. Immunology. (2010) 129:446–54. doi: 10.1111/j.1365-2567.2009.03186.x, PMID: 20002789 PMC2826689

[29] Tchakoute CT Sainani KL Osawe S Datong P Kiravu A Rosenthal KL. Breastfeeding mitigates the effects of maternal hiv on infant infectious morbidity in the option B+ Era. AIDS. (2018) 32:2383–91. doi: 10.1097/qad.0000000000001974, PMID: 30134300

[30] Eckard AR Kirk SE Hagood NL. Contemporary issues in pregnancy (and offspring) in the current hiv era. Curr HIV/AIDS Rep. (2019) 16:492–500. doi: 10.1007/s11904-019-00465-2, PMID: 31630334 PMC6938215

[31] Ikumi NM Matjila M Gray CM Anumba D Pillay K. Placental pathology in women with hiv. Placenta. (2021) 115:27–36. doi: 10.1016/j.placenta.2021.09.006, PMID: 34537469

[32] Ikumi NM Pillay K Tilburgs T Malaba TR Dzanibe S Enninga EAL. T-cell homeostatic imbalance in placentas from women with human immunodeficiency virus in the absence of vertical transmission. J Infect Dis. (2021) 224:S670–S82. doi: 10.1093/infdis/jiab192, PMID: 33880544 PMC8883807

[33] Yampolsky M Shlakhter O Deng D Kala S Walmsley SL Murphy KE. Exploring the impact of hiv infection and antiretroviral therapy on placenta morphology. Placenta. (2021) 104:102–9. doi: 10.1016/j.placenta.2020.12.004, PMID: 33310298

[34] Ikumi NM Malaba TR Pillay K Cohen MC Madlala HP Matjila M. Differential impact of antiretroviral therapy initiated before or during pregnancy on placenta pathology in hiv-positive women. AIDS. (2021) 35:717–26. doi: 10.1097/qad.0000000000002824, PMID: 33724257 PMC8630811

[35] Richter E Bornemann L Korencak M Alter G Schuster M Esser S. Reduction of cd8 T cell functionality but not inhibitory capacity by integrase inhibitors. J Virol. (2022) 96:5. doi: 10.1128/jvi.01730-21, PMID: 35019724 PMC8906395

[36] Schoeman JC Moutloatse GP Harms AC Vreeken RJ Scherpbier HJ Van Leeuwen L. Fetal metabolic stress disrupts immune homeostasis and induces proinflammatory responses in human immunodeficiency virus type 1– and combination antiretroviral therapy–exposed infants. J Infect Dis. (2017) 216:436–46. doi: 10.1093/infdis/jix291, PMID: 28633455 PMC5853663

[37] Ministry of Health M. Malawi hiv subnational estimates (Naomi model 2023) - the document management system . Available online at: https://dms.hiv.health.gov.mw/dataset/malawi-hiv-subnational-estimates-naomi-model-2023 (Accessed July 9, 2025).

[38] Nampota-Nkomba N Buchwald A Nyirenda OM Mkandawire FA Masonga R Meja S. Association between maternal hiv and adverse birth outcomes in the era of universal antiretroviral therapy in Malawi. JAIDS J Acquired Immune Deficiency Syndromes. (2025). doi: 10.1097/qai.0000000000003685, PMID: 40243285 PMC12353428

[39] Hsu H Boudova S Mvula G Divala TH Mungwira RG Harman C. Prolonged pd1 expression on neonatal vdelta2 lymphocytes dampens proinflammatory responses: role of epigenetic regulation. J Immunol. (2016) 197:1884–92. doi: 10.4049/jimmunol.1600284, PMID: 27474072 PMC4992653

[40] Hsu H Boudova S Mvula G Divala TH Rach D Mungwira RG. Age-related changes in pd-1 expression coincide with increased cytotoxic potential in Vδ2 T cells during infancy. Cell Immunol. (2021) 359:104244. doi: 10.1016/j.cellimm.2020.104244, PMID: 33248366 PMC7811364

[41] Gumperz JE Miyake S Yamamura T Brenner MB. Functionally distinct subsets of cd1d-restricted natural killer T cells revealed by cd1d tetramer staining. J Exp Med. (2002) 195:625–36. doi: 10.1084/jem.20011786, PMID: 11877485 PMC2193772

[42] Montoya CJ Pollard D Martinson J Kumari K Wasserfall C Mulder CB. Characterization of human invariant natural killer T subsets in health and disease using a novel invariant natural killer T cell-clonotypic monoclonal antibody, 6b11. Immunology. (2007) 122:1–14. doi: 10.1111/j.1365-2567.2007.02647.x, PMID: 17662044 PMC2265989

[43] den Braanker H Bongenaar M Lubberts E. How to prepare spectral flow cytometry datasets for high dimensional data analysis: A practical workflow. Front Immunol. (2021) 12:768113. doi: 10.3389/fimmu.2021.768113, PMID: 34868024 PMC8640183

[44] Ferrer-Font L Small SJ Lewer B Pilkington KR Johnston LK Park LM. Panel optimization for high-dimensional immunophenotyping assays using full-spectrum flow cytometry. Curr Protoc. (2021) 1. doi: 10.1002/cpz1.222, PMID: 34492732

[45] Herzenberg LA Tung J Moore WA Herzenberg LA Parks DR. Interpreting flow cytometry data: A guide for the perplexed. Nat Immunol. (2006) 7:681–5. doi: 10.1038/ni0706-681, PMID: 16785881

[46] Liechti T Weber LM Ashhurst TM Stanley N Prlic M Van Gassen S. An updated guide for the perplexed: cytometry in the high-dimensional era. Nat Immunol. (2021) 22:1190–7. doi: 10.1038/s41590-021-01006-z, PMID: 34489590

[47] Rach D. Luciernaga: tools for the evaluation of spectral flow cytometry (Sfc) unmixing controls. (2025) v0.99.4 GitHub. Available at: https://github.com/DavidRach/Coereba (Accessed July 9, 2025).

[48] Hsu H Zanettini C Coker M Boudova S Rach D Mvula G. Concomitant assessment of pd-1 and cd56 expression identifies subsets of resting cord blood Vδ2 T cells with disparate cytotoxic potential. Cell Immunol. (2024) 395-396:104797. doi: 10.1016/j.cellimm.2023.104797, PMID: 38157646

[49] Davey MS Willcox CR Hunter S Kasatskaya SA Remmerswaal EBM Salim M. The human Vδ2+ T-cell compartment comprises distinct innate-like Vγ9+ and adaptive Vγ9- subsets. Nat Commun. (2018) 9:1760. doi: 10.1038/s41467-018-04076-0, PMID: 29720665 PMC5932074

[50] Finak G Jiang W Gottardo R. Cytoml for cross-platform cytometry data sharing. Cytometry A. (2018) 93:1189–96. doi: 10.1002/cyto.a.23663, PMID: 30551257 PMC6443375

[51] Finak GJM. Flowworkspace: infrastructure for representing and interacting with gated and ungated cytometry data sets. (2025) Bioconductor. doi: 10.18129/B9.bioc.flowWorkspace (Accessed July 9, 2025).

[52] Emmaneel A Quintelier K Sichien D Rybakowska P Maranon C Alarcon-Riquelme ME. Peacoqc: peak-based selection of high quality cytometry data. Cytometry A. (2022) 101:325–38. doi: 10.1002/cyto.a.24501, PMID: 34549881 PMC9293479

[53] Pedersen CB Dam SH Barnkob MB Leipold MD Purroy N Rassenti LZ. Cycombine Allows for Robust Integration of Single-Cell Cytometry Datasets within and across Technologies. Nat Commun. (2022) 13:1698. doi: 10.1038/s41467-022-29383-5, PMID: 35361793 PMC8971492

[54] Quintelier KLA Willemsen M Bosteels V Aerts J Saeys Y Van Gassen S. Cytonorm 2.0: A flexible normalization framework for cytometry data without requiring dedicated controls. Cytometry A. (2025) 107:69–87. doi: 10.1002/cyto.a.24910, PMID: 39871681

[55] Van Gassen S Gaudilliere B Angst MS Saeys Y Aghaeepour N. Cytonorm: A normalization algorithm for cytometry data. Cytometry Part A. (2020) 97:268–78. doi: 10.1002/cyto.a.23904, PMID: 31633883 PMC7078957

[56] Rach D. Coereba: dichotomized clustering for spectral flow cytometry. (2025) v0.99.1 GitHub. Available at: https://github.com/DavidRach/Coereba (Accessed July 9, 2025).

[57] Finak G Frelinger J Jiang W Newell EW Ramey J Davis MM. Opencyto: an open source infrastructure for scalable, robust, reproducible, and automated, end-to-end flow cytometry data analysis. PloS Comput Biol. (2014) 10:e1003806. doi: 10.1371/journal.pcbi.1003806, PMID: 25167361 PMC4148203

[58] Wang Y HH Rudin C Shaposhnik Y. Understanding how dimension reduction tools work: an empirical approach to deciphering T-sne, umap, trimap, and pacmap for data visualization. J Mach Learn Res. (2021) 22:1–73.

[59] Belkina AC Ciccolella CO Anno R Halpert R Spidlen J Snyder-Cappione JE. Automated optimized parameters for T-distributed stochastic neighbor embedding improve visualization and analysis of large datasets. Nat Commun. (2019) 10:5415. doi: 10.1038/s41467-019-13055-y, PMID: 31780669 PMC6882880

[60] Kobak D Linderman GC. Initialization is critical for preserving global data structure in both T-sne and umap. Nat Biotechnol. (2021) 39:156–7. doi: 10.1038/s41587-020-00809-z, PMID: 33526945

[61] Moon KR van Dijk D Wang Z Gigante S Burkhardt DB Chen WS. Visualizing structure and transitions in high-dimensional biological data. Nat Biotechnol. (2019) 37:1482–92. doi: 10.1038/s41587-019-0336-3, PMID: 31796933 PMC7073148

[62] Wickham H Averick M Bryan J Chang W McGowan L François R. Welcome to the tidyverse. J Open Source Software. (2019) 4(43):1686. doi: 10.21105/joss.01686

[63] Iannone R CJ Schloerke B Hughes E Lauer A Seo J Brevoort K. Gt: easily create presentation-ready display tables. (2025) CRAN. doi: 10.32614/CRAN.package.gt (Accessed July 9, 2025).

[64] Seiler C Ferreira AM Kronstad LM Simpson LJ Le Gars M Vendrame E. Cytoglmm: conditional differential analysis for flow and mass cytometry experiments. BMC Bioinf. (2021) 22:137. doi: 10.1186/s12859-021-04067-x, PMID: 33752595 PMC7983283

[65] R Core Team. R: A language and environment for statistical computing. Vienna, Austria: R Foundation for Statistical Computing (2024).

[66] Wuertz D ST Chalabi Y Boshnakov GN. Fbasics: rmetrics - markets and basic statistics. (2024) CRAN. doi: 10.32614/CRAN.package.fBasics (Accessed July 9, 2025).

[67] Dunne MR Mangan BA Madrigal-Estebas L Doherty DG. Preferential th1 cytokine profile of phosphoantigen-stimulated human VΓ9vΔ2 T cells. Mediators Inflammation. (2010) 2010:1–11. doi: 10.1155/2010/704941, PMID: 21403900 PMC3043297

[68] Moens E Brouwer M Dimova T Goldman M Willems F Vermijlen D. Il-23r and tcr signaling drives the generation of neonatal vgamma9vdelta2 T cells expressing high levels of cytotoxic mediators and producing ifn-gamma and il-17. J Leukoc Biol. (2011) 89:743–52. doi: 10.1189/jlb.0910501, PMID: 21330350

[69] Morita CT Parker CM Brenner MB Band H. Tcr usage and functional capabilities of human gamma delta T cells at birth. J Immunol. (1994) 153:3979–88. doi: 10.4049/jimmunol.153.9.3979, PMID: 7930606

[70] Tan L Fichtner AS Bruni E Odak I Sandrock I Bubke A. A fetal wave of human type 3 effector Γδ Cells with restricted tcr diversity persists into adulthood. Sci Immunol. (2021) 6:eabf0125. doi: 10.1126/sciimmunol.abf0125, PMID: 33893173

[71] Ryan PL Sumaria N Holland CJ Bradford CM Izotova N Grandjean CL. Heterogeneous yet stable vdelta2(+) T-cell profiles define distinct cytotoxic effector potentials in healthy human individuals. Proc Natl Acad Sci U.S.A. (2016) 113:14378–83. doi: 10.1073/pnas.1611098113, PMID: 27911793 PMC5167212

[72] Constant P Davodeau F Peyrat M-A Poquet Y Puzo G Bonneville M. Stimulation of human Γδ T cells by nonpeptidic mycobacterial ligands. Science. (1994) 264:267–70. doi: 10.1126/science.8146660, PMID: 8146660

[73] Gober H-JR Kistowska M Angman L Jenoü P Mori L De Libero G. Human T cell receptor Γδ Cells recognize endogenous mevalonate metabolites in tumor cells. J Exp Med. (2003) 197:163–8. doi: 10.1084/jem.20021500, PMID: 12538656 PMC2193814

[74] Tanaka Y Morita CT Tanaka Y Nieves E Brenner MB Bloom BR. Natural and synthetic non-peptide antigens recognized by human Γδ T cells. Nature. (1995) 375:155–8. doi: 10.1038/375155a0, PMID: 7753173

[75] Eberl M Hintz M Reichenberg A Kollas A-K Wiesner J Jomaa H. Microbial isoprenoid biosynthesis and human Γδ T cell activation. FEBS Lett. (2003) 544:4–10. doi: 10.1016/s0014-5793(03)00483-6, PMID: 12782281

[76] Hintz M Reichenberg A Altincicek B Bahr U Gschwind RM Kollas A-K. Identification of (E)-4-hydroxy-3-methyl-but-2-enyl pyrophosphate as a major activator for human Γδ T cells in escherichia coli. FEBS Lett. (2001) 509:317–22. doi: 10.1016/s0014-5793(01)03191-x, PMID: 11741609

[77] Jomaa H Feurle J Lühs K Kunzmann V Tony H-P Herderich M. Vî³9/vî´2 T cell activation induced by bacterial low molecular mass compounds depends on the 1-deoxy-D-xylulose 5-phosphate pathway of isoprenoid biosynthesis. FEMS Immunol Med Microbiol. (1999) 25:371–8. doi: 10.1111/j.1574-695x.1999.tb01362.x, PMID: 10497868

[78] Shen Y Zhou D Qiu L Lai X Simon M Shen L. Adaptive immune response of Vγ2vδ2^+^ T cells during mycobacterial infections. Science. (2002) 295:2255–8. doi: 10.1126/science.1068819, PMID: 11910108 PMC2872146

[79] Le Roux SM Donald KA Kroon M Phillips TK Lesosky M Esterhuyse L. Hiv viremia during pregnancy and neurodevelopment of hiv-exposed uninfected children in the context of universal antiretroviral therapy and breastfeeding. Pediatr Infect Dis J. (2019) 38:70–5. doi: 10.1097/inf.0000000000002193, PMID: 30234792 PMC7363962

[80] Reantragoon R Corbett AJ Sakala IG Gherardin NA Furness JB Chen Z. Antigen-loaded mr1 tetramers define T cell receptor heterogeneity in mucosal-associated invariant T cells. J Exp Med. (2013) 210:2305–20. doi: 10.1084/jem.20130958, PMID: 24101382 PMC3804952

[81] Swarbrick GM Gela A Cansler ME Null MD Duncan RB Nemes E. Postnatal expansion, maturation, and functionality of mr1t cells in humans. Front Immunol. (2020) 11:556695. doi: 10.3389/fimmu.2020.556695, PMID: 33042140 PMC7524872

[82] Baines KJ West RC. Sex differences in innate and adaptive immunity impact fetal, placental, and maternal healthdagger. Biol Reprod. (2023) 109:256–70. doi: 10.1093/biolre/ioad072, PMID: 37418168

[83] Nolan JP. The evolution of spectral flow cytometry. Cytometry Part A. (2022) 101:812–7. doi: 10.1002/cyto.a.24566, PMID: 35567367

