# Supplementary material for: Cord blood innate-like T cell responses in neonates born to healthy women and women living with HIV
Source: Front Immunol. 2025 Aug 22;16:1628145. doi: 10.3389/fimmu.2025.1628145 (PMC12412222; doi:10.3389/fimmu.2025.1628145)
Supplement: Supplementary file 1 [file DataSheet1.pdf]

Supplementary Appendix

Cord Blood Innate-like T cell responses in neonates born to healthy women and women living with HIV

Rach D et al.

Data and Code Availability

All SFC .fcs files generated for this paper are available via the ImmPort repository (study accession SDY3080). The SFC data consists of the raw .fcs files for fully stained samples with the matched unstained and single-color unmixing controls. We have attempted to format the metadata in accordance with both ImmPort and MIFlow guidelines.

The visualized quality control data for the 5-laser Cytek Aurora on which the specimens were acquired in 2023 is available via the UMGCC FCSS dashboard (https://umgccfcss.github.io/Aurora5L/)

A full list of R packages and the R code used for the data visualizations and statistical analysis for this paper can be found at https://github.com/DavidRach/CordBloodILT, along with additional relevant and insightful information about laboratory protocols, instrumental quality, etc.

The Luciernaga (https://github.com/DavidRach/Luciernaga) and Coereba (https://github.com/DavidRach/Coereba) R packages are available on GitHub, under the free AGPL-3.0-or-later copyleft license.

Supplementary Table 1. SFC Antibodies

Marker	Alternate Name	Clone	Isotype	Fluorophore	Manufacturer	Catalog Number	Temperature (°C)	Location
CD62L		SK11	Mouse IgG2a, k	BUV395	BD Biosciences	565219	4°	Surface
CD8	CD8a	RPA-T8	Mouse IgG1, k	BUV496	BD Biosciences	612942	4°	Surface
CD69		FN50	Mouse IgG1, k	BUV563	BD Biosciences	748764	4°, RT	Surface, IC
CD194	CCR4	1G1	Mouse IgG1, k	BUV615	BD Biosciences	613000	37°	Surface
Vδ2		B6	Mouse IgG2, k	BUV661	BD Biosciences	750056	4°, RT	Surface, IC
CD183	CXCR3	1C6/CXCR3	Mouse IgG1, k	BUV737	BD Biosciences	741866	4°	Surface
CD4		SK3	Mouse IgG1, k	BUV805	BD Biosciences	612887	37°, RT	Surface, IC
CD127	IL-7Ra	A019D5	Mouse IgG1, k	BV421	BioLegend	351310	37°	Surface
CD14		M5E2	Mouse IgG2a, k	Pacific Blue	BioLegend	301828	4°	Surface
CD19		SJ25C1	Mouse IgG1, k	Pacific Blue	BioLegend	363036	4°	Surface
CD161		HP-3G10	Mouse IgG1, k	BV480	BD Biosciences	748279	37°	Surface
CD45RA		HI100	Mouse IgG2b, k	BV510	BioLegend	304142	4°	Surface
CD56		5.1H11	Mouse IgG1, k	BV605	BioLegend	362538	37°, RT	Surface, IC
CD197	CCR7	G043H7	Mouse IgG2a, k	BV650	BioLegend	353234	37°, RT	Surface, IC
CD7		M-T701	Mouse IgG1, k	BV711	BD Biosciences	564018	37°	Surface
IFNγ		B27	Mouse IgG1, k	BV750	BD Biosciences	566357	RT	Intracellular
CD196	CCR6	11A9	Mouse IgG1, k	BV786	BD Biosciences	563704	37°	Surface
CD3		SK7	Mouse IgG1, k	Spark Blue 550	BioLegend	344852	4°, RT	Surface, IC

Supplementary Table 1. SFC Antibodies

Marker	Alternate Name	Clone	Isotype	Fluorophore	Manufacturer	Catalog Number	Temperature (°C)	Location
hCD1d PBS-57			Tetramer	Alexa Fluor 488	NIH Tetramer Core		RT	Surface
Vα24Jα18		6B11	Mouse IgG1, k	FITC	BioLegend	342906	RT	Surface
CD314	NKG2D	1D11	Mouse IgG1, k	PE	BioLegend	320806	37°, RT	Surface, IC
CD26		BA5b	Mouse IgG2a, k	PerCP-Cy5.5	BioLegend	302716	RT	Intracellular
CD25	IL-2Ra	M-A251	Mouse IgG1, k	PE-Cy5	BD Biosciences	555433	37°, RT	Surface
TNFα		MAb11	Mouse IgG1, k	PE-Dazzle 594	BioLegend	502946	RT	Intracellular
CD279	PD1	PD1.3.1.3	Mouse IgG2b	PE-Vio 770	Miltenyi Biotec	130-117-698	37°, RT	Surface, IC
CD16	FcyRIII	3G8	Mouse IgG1, k	APC	BioLegend	302012	4°	Surface
hMR1 5-OP-RU			Tetramer	Alexa Fluor 647	NIH Tetramer Core		RT	Surface
Vα7.2		3C10	Mouse IgG1, k	Alexa Fluor 647	BioLegend	351726	RT	Surface
CD107a		H4A3	Mouse IgG1, k	APC-R700	BD Biosciences	565184	37°	Surface
CD27		O323	Mouse IgG1, k	APC-Fire 750	BioLegend	302846	37°	Surface
CD38		HIT2	Mouse IgG1, k	APC-Fire 810	BioLegend	303550	37°	Surface
CD3*		UCHT1	Mouse IgG1, k	Alexa Fluor 488	BioLegend	300415	4°	Surface
CD3*		OKT3	Mouse IgG2a, k	Alexa Fluor 647	BioLegend	317312	4°	Surface

RT = Stained at room temperature; 4°, RT = Antibody incubated at 4°C for surface staining and at room temperature for intracellular staining; 37°, RT = Antibody incubated at 37°C for surface staining and at room temperature for intracellular staining; IC = Intracellular. *Only used as unmixing controls for tetramers on respective fluorophores.

Supplementary Table 2. CFC Antibodies

Marker	Alternate Name	Clone	Isotype	Fluorophore	Manufacturer	Catalog Number	Panel	Location
CD279	PD1	EH12.2H7	Mouse IgG1, k	BV421	BioLegend	329920	CK0, CK17, 1, 4b	Surface
Vδ2		B6	Mouse IgG1, k	BV421	BioLegend	331428	2	Surface
Viability				Zombie Aqua Fixable Viability dye	BioLegend	423101	CK0, CK17, 1, 4b	Surface
CD3		UCHT1	Mouse IgG1, k	BV510	BioLegend	300448	2	Surface
CD56	NCAM	5.1H11	Mouse IgG1, k	BV650	BioLegend	362532	CK17, 4b	Surface
CD27		O323	Mouse IgG1, k	BV650	BioLegend	302828	CK0	Surface
CD16		3G8	Mouse IgG1, k	BV650	BioLegend	302042	1	Surface
CD314	NKG2D	1D11	Mouse IgG1, k	Biotin	BioLegend	320804	2	Surface
Streptavidin				BV650	BioLegend	405232	2	Surface
CD107a	LAMP-1	H4A3	Mouse IgG1, k	Alexa Fluor 488	BioLegend	328610	CK17	Surface
Perforin		dG9	Mouse IgG2b, k	Alexa Fluor 488	BioLegend	308108	2, 4b	Intracellular
Vδ2		B6	Mouse IgG1, k	FITC	BioLegend	331406	CK0, 1	Surface
CD3		OKT3	Mouse IgG2a, k	PerCP-eFluor 710	Invitrogen	46-0037-42	CK0, 4b	Surface
CD25		CD25-4E3	Mouse IgG2b, k	PerCP-eFluor 710	Invitrogen	46-0257-42	1	Surface
TNFα		MAb11	Mouse IgG1, k	PerCP-Cy5.5	BioLegend	502926	CK17	Intracellular
CD56	NCAM	TULY56	Mouse IgG1, k	PerCP-eFluor 710	Invitrogen	46-0566-42	2	Surface
Granzyme B		GB11	Mouse IgG1, k	PE	BD Biosciences	561142	4b	Intracellular
CD56		5.1H11	Mouse IgG1, k	PE	BioLegend	362508	CK0	Surface

Supplementary Table 2. CFC Antibodies

Marker	Alternate Name	Clone	Isotype	Fluorophore	Manufacturer	Catalog Number	Panel	Location
CD28		"15E8"	Mouse IgG1, k	PE	Miltenyi Biotec	130-126-172	1	Surface
Vδ2		B6	Mouse IgG1, k	PE	BioLegend	331408	CK17	Surface
CD279	PD1	EH12.2H7	Mouse IgG1, k	PE	BioLegend	329906	2	Surface
CD27		O323	Mouse IgG1, k	PE-Dazzle 594	BioLegend	302844	1	Surface
CD159a	NKG2A	REA110	Recombinant human IgG1	PE-Vio 770	Miltenyi Biotec	130-113-567	2, 4b	Surface
IFNγ		45-15	Mouse IgG1, k	PE-Vio 770	Miltenyi Biotec	130-113-494	CK0, CK17	Intracellular
CD3		BW264/58	Mouse IgG2a, k	PE-Vio 770	Miltenyi Biotec	130-113-130	1	Surface
Vδ2		B6	Mouse IgG1, k	APC	BioLegend	331418	4b	Surface
CD27		O323	Mouse IgG1, k	APC	BioLegend	302810	CK17	Surface
TNFα		MAb11	Mouse IgG1, k	Alexa Fluor 647	BioLegend	502916	CK0	Intracellular
Vδ1		REA173	Recombinant human IgG1	APC	Miltenyi Biotec	130-118-968	1, 2	Surface
CD16		3G8	Mouse IgG1, k	APC-Fire 750	BioLegend	302060	4b	Surface
CD45RO		UCHL1	Mouse IgG2a, k	APC-Fire 750	BioLegend	304250	CK0, CK17	Surface
CD45RA		HI100	Mouse IgG2b, k	APC-Fire 750	BioLegend	304152	1	Surface
Viability				BD Horizon 780	BD Biosciences	565388	2	Surface
CD3*		UCHT1	Mouse IgG1, k	BV421	BioLegend	300434		Surface
CD3*		UCHT1	Mouse IgG1, k	BV510	BioLegend	300448		Surface
CD3*		OKT3	Mouse IgG2a, k	BV650	BioLegend	317324		Surface
CD3*		UCHT1	Mouse IgG1, k	FITC	BioLegend	300406		Surface

Supplementary Table 2. CFC Antibodies

Marker	Alternate Name	Clone	Isotype	Fluorophore	Manufacturer	Catalog Number	Panel	Location
CD3*		OKT3	Mouse IgG2a, k	PerCP-eFluor 710	Invitrogen	46-0037-42		Surface
CD3*		UCHT1	Mouse IgG1, k	PE	BioLegend	300441		Surface
CD3*		UCHT1	Mouse IgG1, k	PE-Dazzle 594	BioLegend	300450		Surface
CD3*		BW264/58	Mouse IgG2a, k	PE-Vio 770	Miltenyi Biotec	130-113-130		Surface
CD3*		OKT3	Mouse IgG2a, k	APC	BioLegend	317318		Surface
CD3*		UCHT1	Mouse IgG1, k	APC-Fire 750	BioLegend	300470		Surface

1 = Phenotype, 2 = NK markers, 4b = Cytotoxicity, CK = Cytokine, 0 = *Ex Vivo*, 17 = Post expansion

* Only used for compensation controls

Supplementary Table 3. Generalized Linear Model Results

Marker ~ Infant Sex + HIV/ART Exposure
Separate models for each dependent variable

Tested	Predictor	OR	SE	z-value	p-value	OR [95% CI]	[1]
Vδ2							
NKG2D	Intercept	2.01	0.03	20.62	**< 0.001**	2.01 [1.88–2.15]	***
NKG2D	Sex: Male	0.92	0.03	-2.55	**0.014**	0.92 [0.87–0.98]	*
NKG2D	Exposure: HEU-lo	1.01	0.04	0.20	0.845	1.01 [0.93–1.09]	
NKG2D	Exposure: HEU-hi	0.95	0.04	-1.43	0.158	0.95 [0.88–1.02]	
CD8	Intercept	1.40	0.04	8.18	**< 0.001**	1.40 [1.29–1.52]	***
CD8	Sex: Male	0.90	0.04	-2.61	**0.012**	0.90 [0.84–0.97]	*
CD8	Exposure: HEU-lo	1.01	0.05	0.18	0.855	1.01 [0.92–1.11]	
CD8	Exposure: HEU-hi	0.97	0.05	-0.55	0.582	0.97 [0.89–1.07]	
CD45RA	Intercept	1.93	0.03	18.99	**< 0.001**	1.93 [1.80–2.07]	***
CD45RA	Sex: Male	0.93	0.03	-2.29	**0.026**	0.93 [0.87–0.99]	*
CD45RA	Exposure: HEU-lo	0.99	0.04	-0.13	0.896	0.99 [0.92–1.08]	
CD45RA	Exposure: HEU-hi	0.97	0.04	-0.66	0.509	0.97 [0.90–1.05]	
CD62L	Intercept	1.11	0.02	5.98	**< 0.001**	1.11 [1.07–1.15]	***
CD62L	Sex: Male	1.04	0.02	2.18	**0.034**	1.04 [1.00–1.07]	*
CD62L	Exposure: HEU-lo	1.02	0.02	0.95	0.346	1.02 [0.98–1.06]	
CD62L	Exposure: HEU-hi	1.04	0.02	2.10	**0.04**	1.04 [1.00–1.08]	*
CD25	Intercept	1.36	0.04	7.07	**< 0.001**	1.36 [1.25–1.48]	***
CD25	Sex: Male	1.09	0.04	2.04	**0.046**	1.09 [1.00–1.18]	*
CD25	Exposure: HEU-lo	0.99	0.05	-0.19	0.852	0.99 [0.90–1.10]	
CD25	Exposure: HEU-hi	1.04	0.05	0.74	0.464	1.04 [0.94–1.14]	
MAIT							
PD1	Intercept	1.46	0.04	8.69	**< 0.001**	1.46 [1.34–1.58]	***
PD1	Sex: Male	1.09	0.04	2.00	**0.05**	1.09 [1.00–1.18]	*
PD1	Exposure: HEU-lo	1.03	0.05	0.60	0.554	1.03 [0.93–1.14]	
PD1	Exposure: HEU-hi	0.96	0.05	-0.73	0.468	0.96 [0.88–1.06]	

[1] ***p < 0.001; **p < 0.01; *p < 0.05

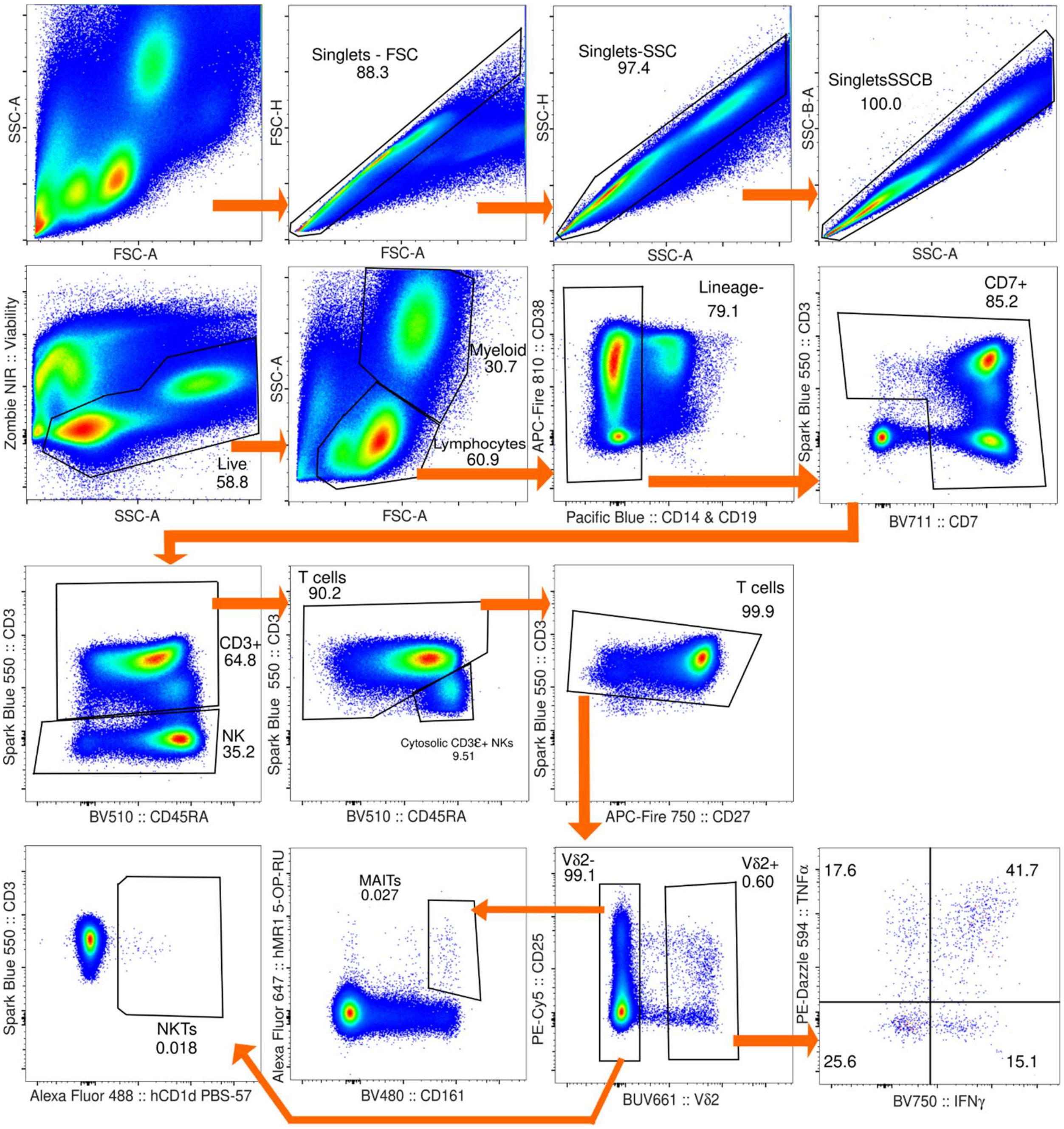

Supplementary Figure 1. Gating strategy for spectral flow cytometry (SFC) panel. The dot plots illustrate the gating strategy used to identify the main ILT nodes for a representative HIV-unexposed (HU) infant, with descending hierarchical gates denoted by arrows. Individual gates and frequency in the parent gate are shown.

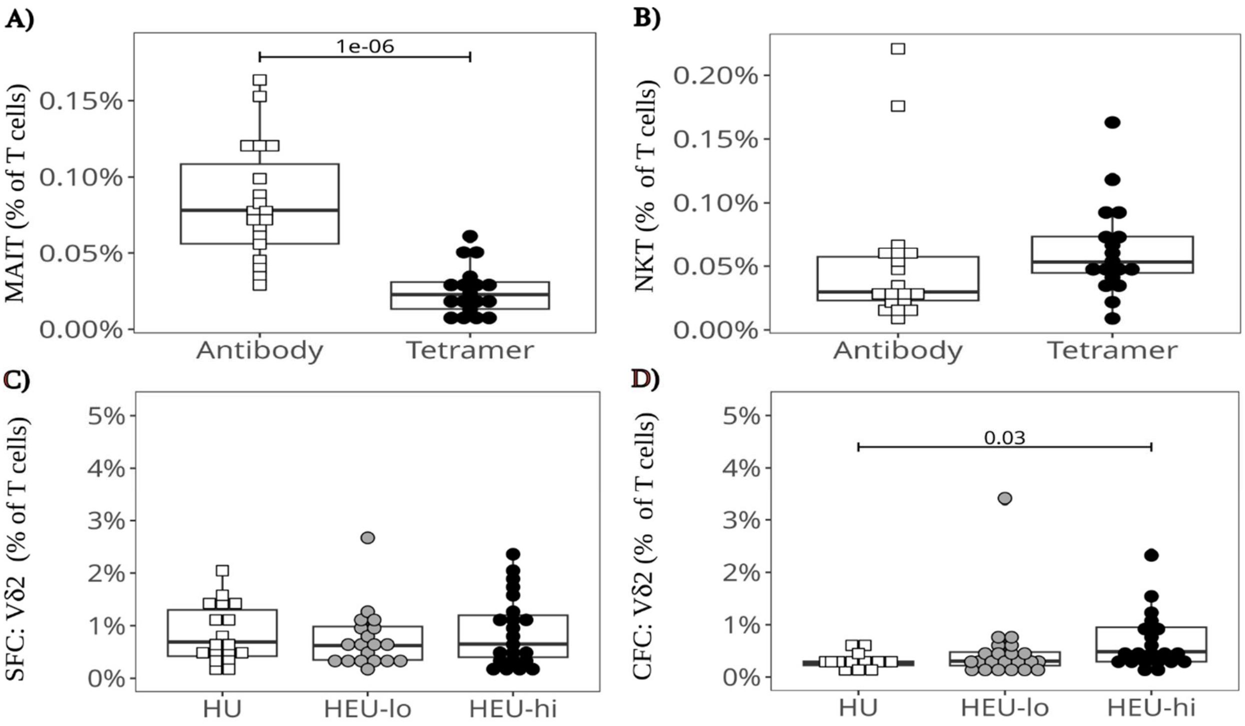

Supplementary Figure 2. Frequency of MAITs and NKTs by antibody versus tetramer staining. The beeswarm plots display the frequency of ILT subsets, with individual symbols representing unique HU specimens, boxplots depicting median and IQR, with whiskers showing the +/- 1.5 IQR range. (A) Frequency of MAITs identified by antibody ($CD161^{hi}$ Vα7.2+) or tetramer ($CD161^{hi}$ hMR1 5-OP-RU+) cells, as percentage of viable T cells. (B) Frequency of NKTs by identified by antibody (Vα24Jα18+ cells) or tetramer (hCD1d PBS-57+) cells as percentage of viable T cells. Frequency of Vδ2s in spectral flow cytometry (SFC) acquired specimens (C) or in a non-overlapping subset of conventional flow cytometry (CFC) acquired specimens (D), as percentage of viable T cells.

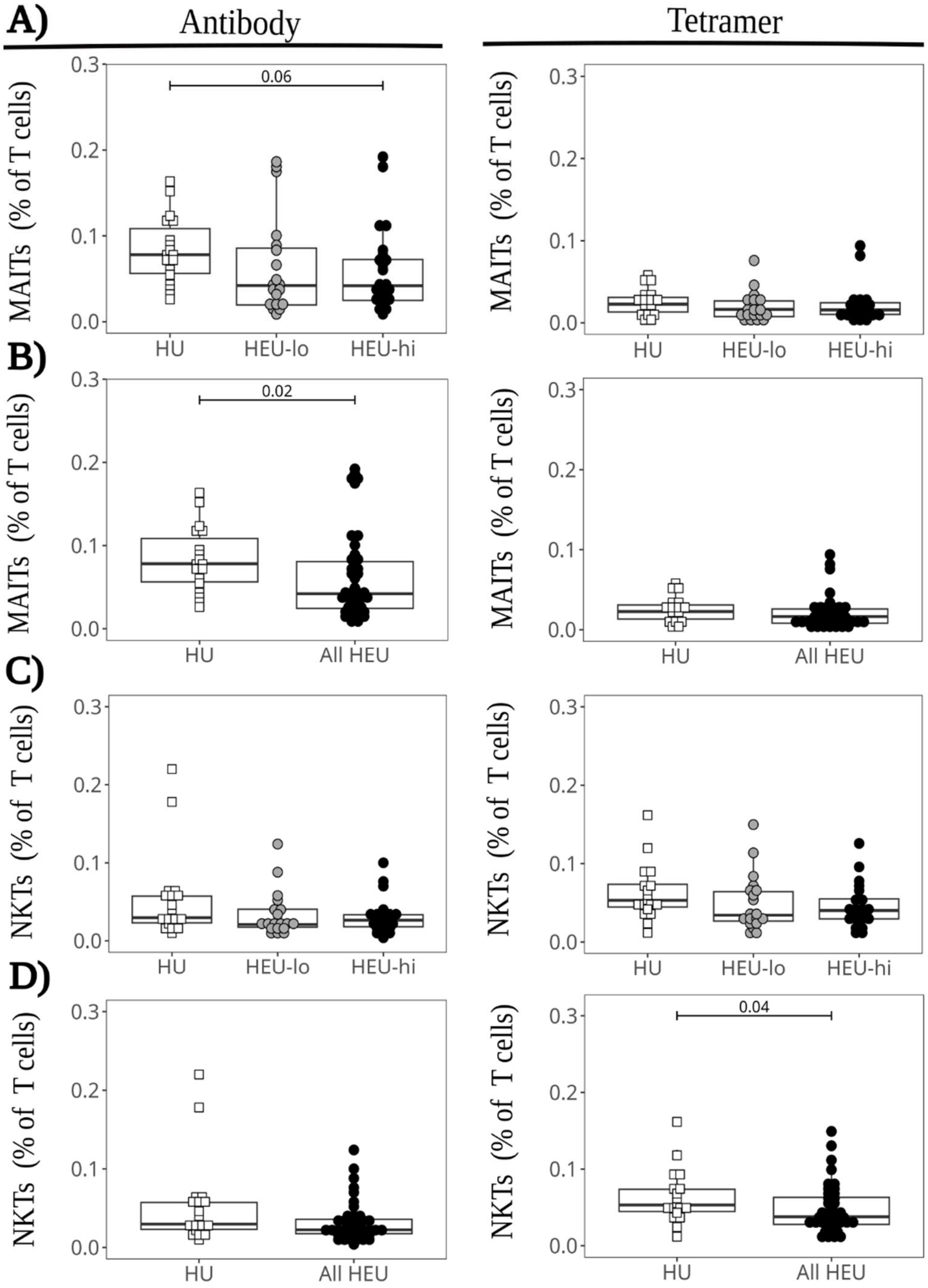

Supplementary Figure 3. Antibody and Tetramer-staining frequency of ILT subsets in HEU infants. The beeswarm plots show the frequency of ILT subsets with individual symbols representing unique specimens, boxplots depicting median and IQR, with whiskers showing the +/- 1.5 IQR range. (A) Frequency of MAITs identified by antibody (CD161^{hi} Vα7.2+, left) or tetramer (CD161^{hi} hMR1 5-OP-RU+, right). (B) Frequency of MAITs identified by antibody (CD161^{hi} Vα7.2+, left) or tetramer (CD161^{hi} hMR1 5-OP-RU+, right) and pooled by HIV-exposure. (C) Frequency of NKTs identified by antibody (Vα24Jα18+, left) or tetramer (hCD1d PBS-57+, right) staining. (D) Frequency of NKTs pooled by identified by antibody (Vα24Jα18+, left) or tetramer (hCD1d PBS-57+, right) staining and pooled by HIV-exposure.

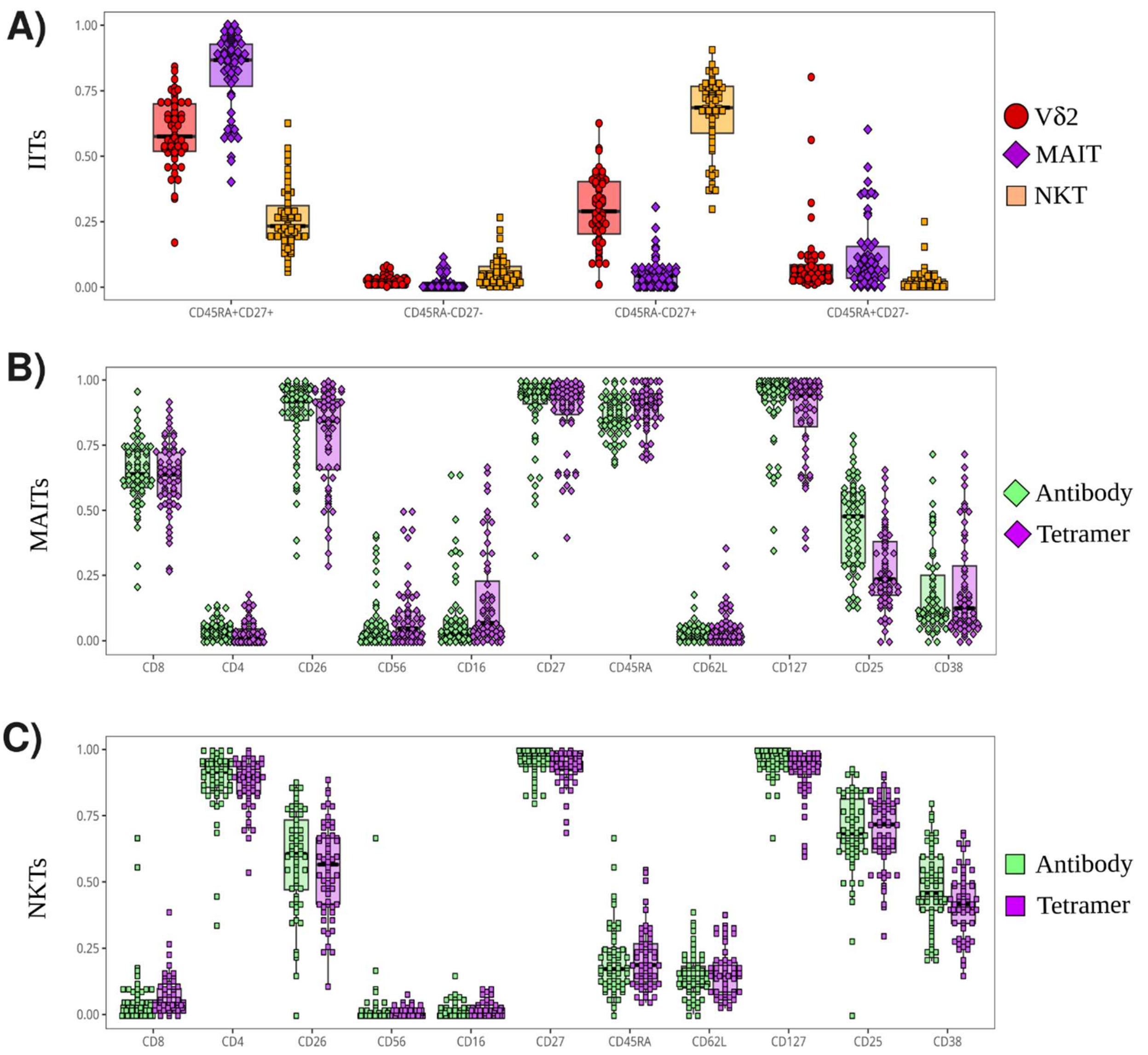

Supplementary Figure 4. Differentiation of Cord Blood ILTs. (A) The beeswarm plot displays the memory differentiation across ILT subsets, with each subset designated by a color and a symbol. Individual symbols represent the individual proportions of cells falling into each differentiation state listed on the x-axis. (B) The beeswarm plots compare the expression of specific markers in MAITs defined by either antibody (CD161^{hi} Vα7.2+) or tetramer (CD161^{hi} hMR1 5-OP-RU+), designated by shape and color. (C) The beeswarm plots compare the expression of specific markers in NKTs identified by either antibody (Vα24Jα18+) or tetramer (hCD1d PBS-57+), designated by shape and color. Symbols represent individual values, boxplots depict medians and IQR, with whiskers showing the +/- 1.5 IQR range.

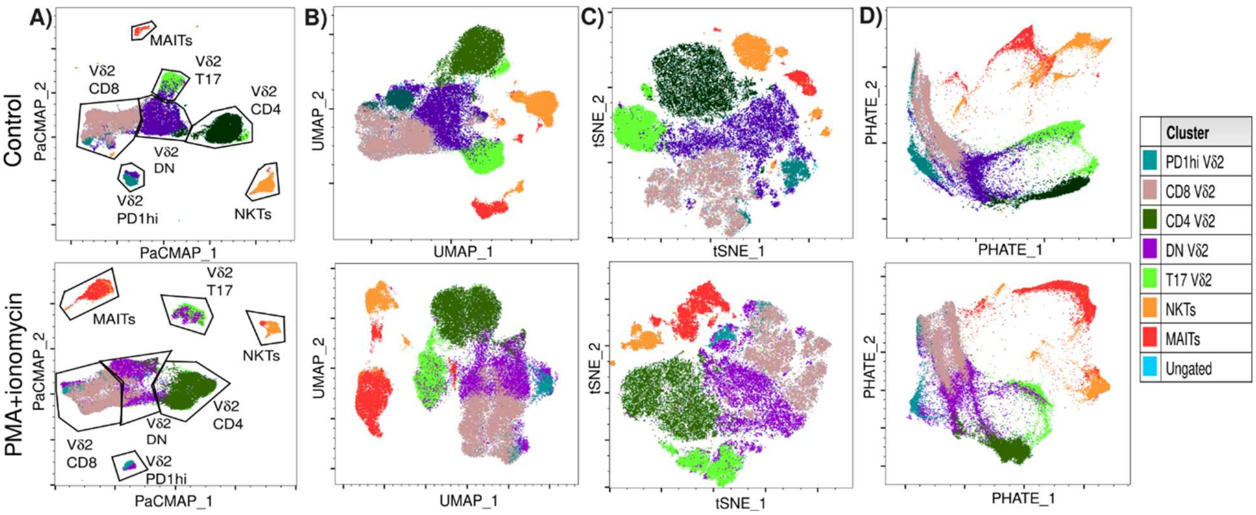

Supplementary Figure 5. Different visualization algorithms identify comparable ILT clusters. The results of multiple visualization algorithms are shown side by side, with the same ILT cell gates (manually drawn in concatenated ILT files) overlaid on the different maps. (A) PaCMAP, (B) UMAP, (C) tSNE, (D) and PHATE results are shown for unstimulated (top) and PMA+ionomycin stimulated ILT.

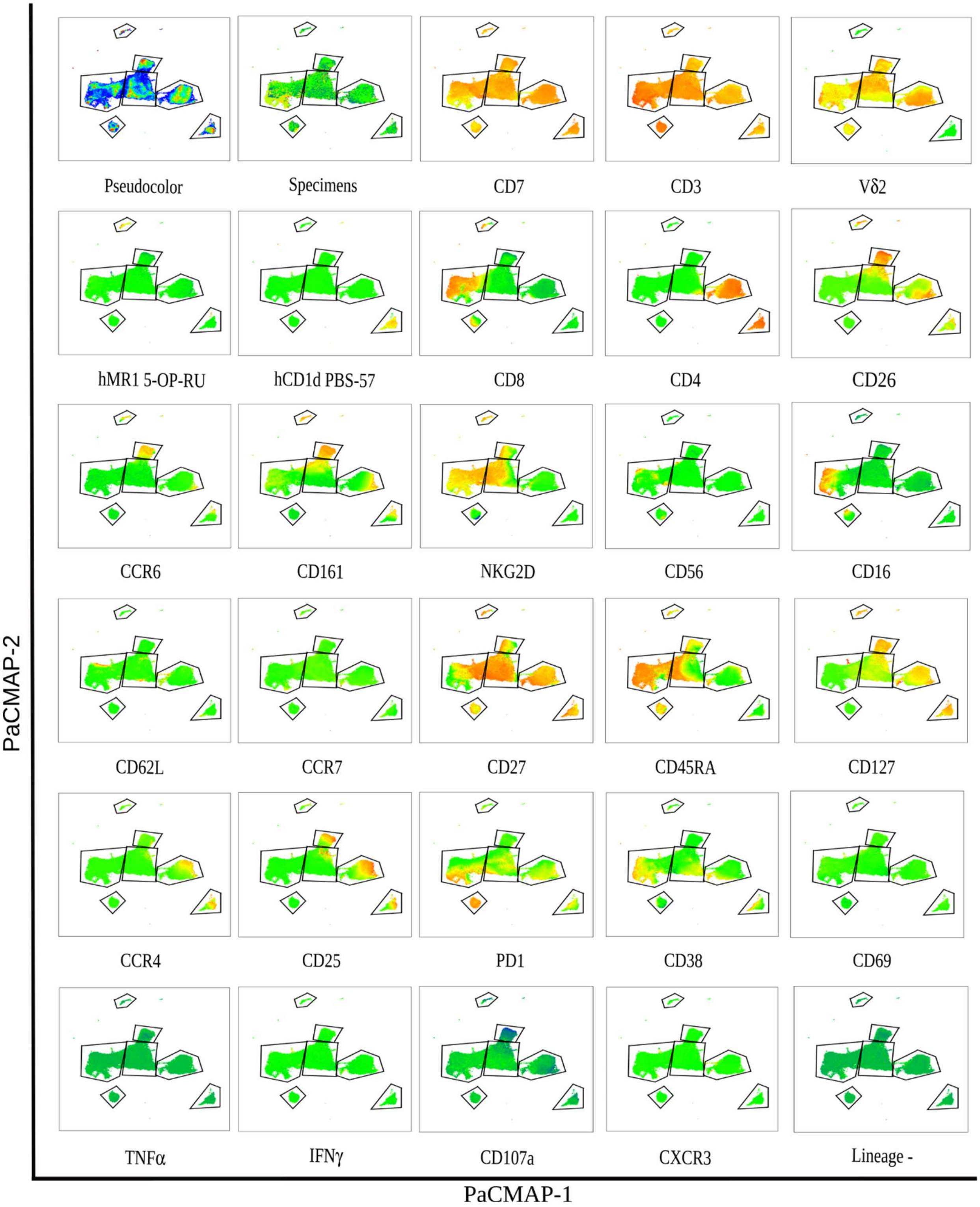

Supplementary Figure 6. Heatmap visualization for individual markers at baseline. Each plot in the grid shows the expression individual markers by heat indexing on the basis of median fluorescent intensity (MFI) for PaCMAP generated ILT subsets in a concatenated file, including Vδ2 cells and tetramer identified MAITs and NKTs at baseline. Scale: green=low, red=high.

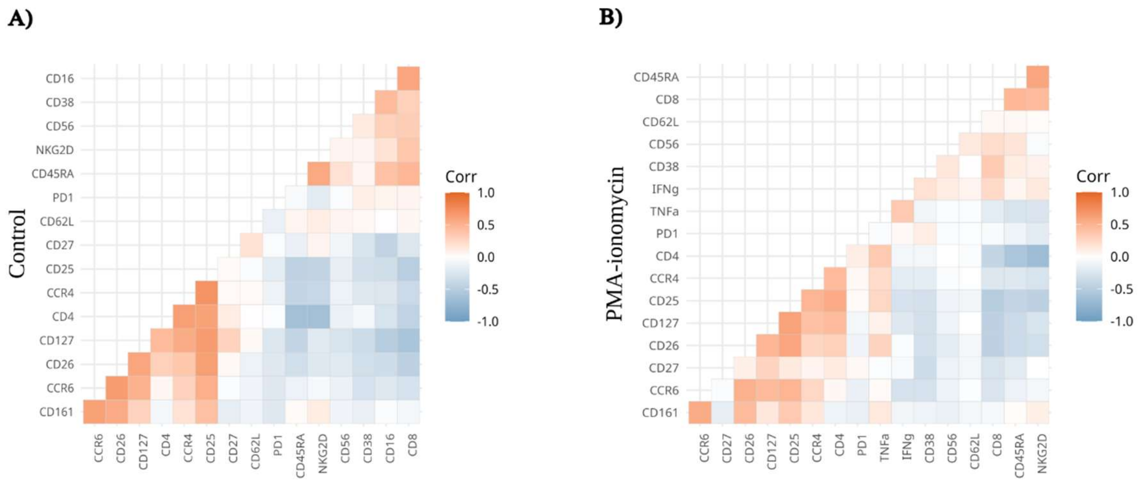

Supplementary Figure 7. Marker Co-expression for Vδ2 cells at baseline and after activation *ex vivo*. Manually gated Vδ2 cells across all HIV-unexposed (HU) specimens were concatenated and marker co-expressions matrices were generated using CytoGLMM R package. The heatmap function indicates the extent of marker co-expression, ranging from low (blue) to high (red). (A) Marker co-expression plot for cord blood Vδ2 cells at baseline. (B) Marker co-expression plot of cord blood Vδ2 cells after activation with PMA+ionomycin.

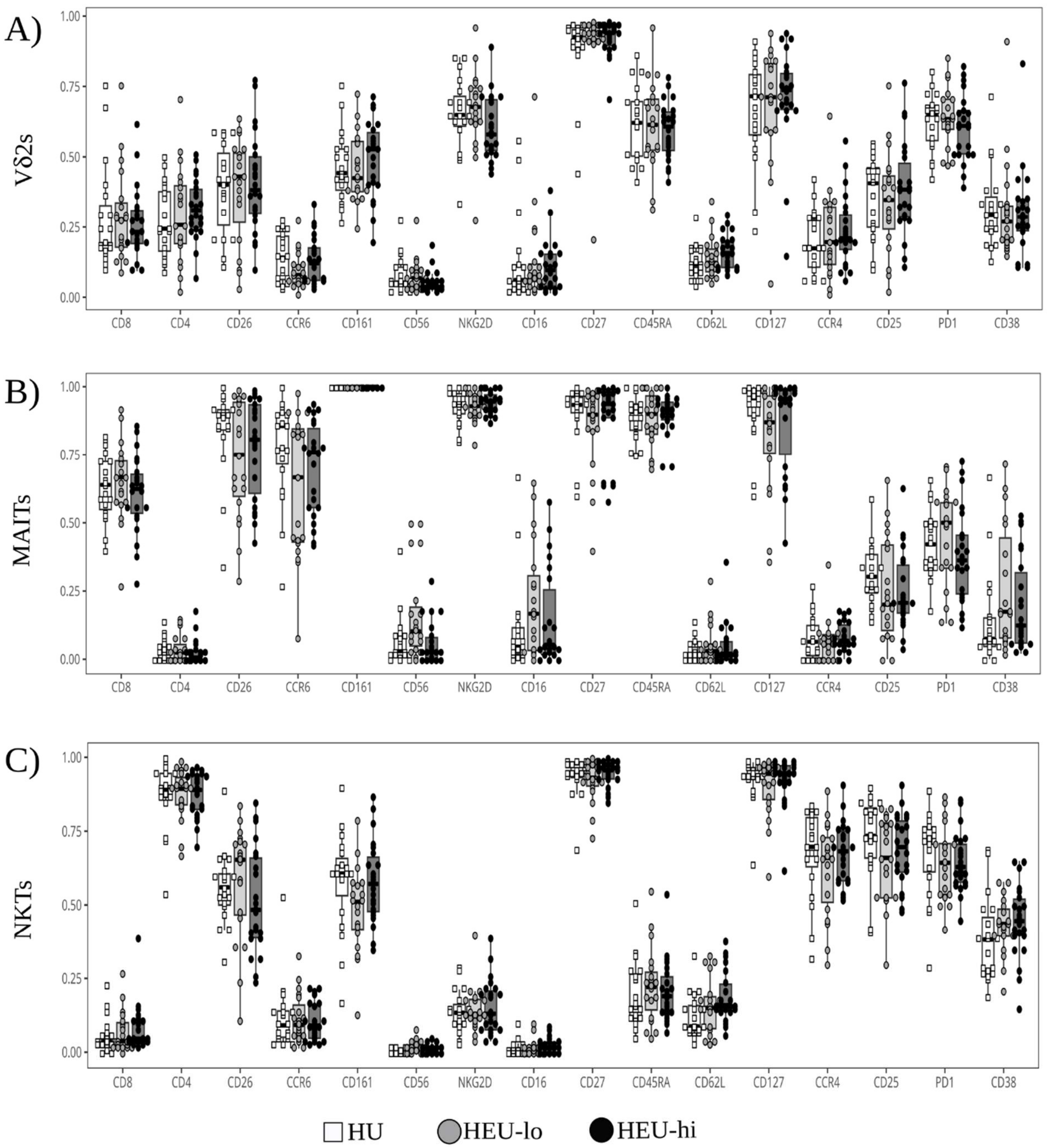

Supplemental Figure 8. Cord blood ILT subsets at baseline are characterized by specific marker expression profiles. (A) The beeswarm plots show the proportion of cells expressing individual markers listed on the x-axis for Vδ2 (A), MAIT (B) and NKT (C) cells, respectively. The symbols show individual values, boxplots show median and IQR, with whiskers indicating the +/- 1.5 IQR range. Shape and color identify HIV-exposure status.

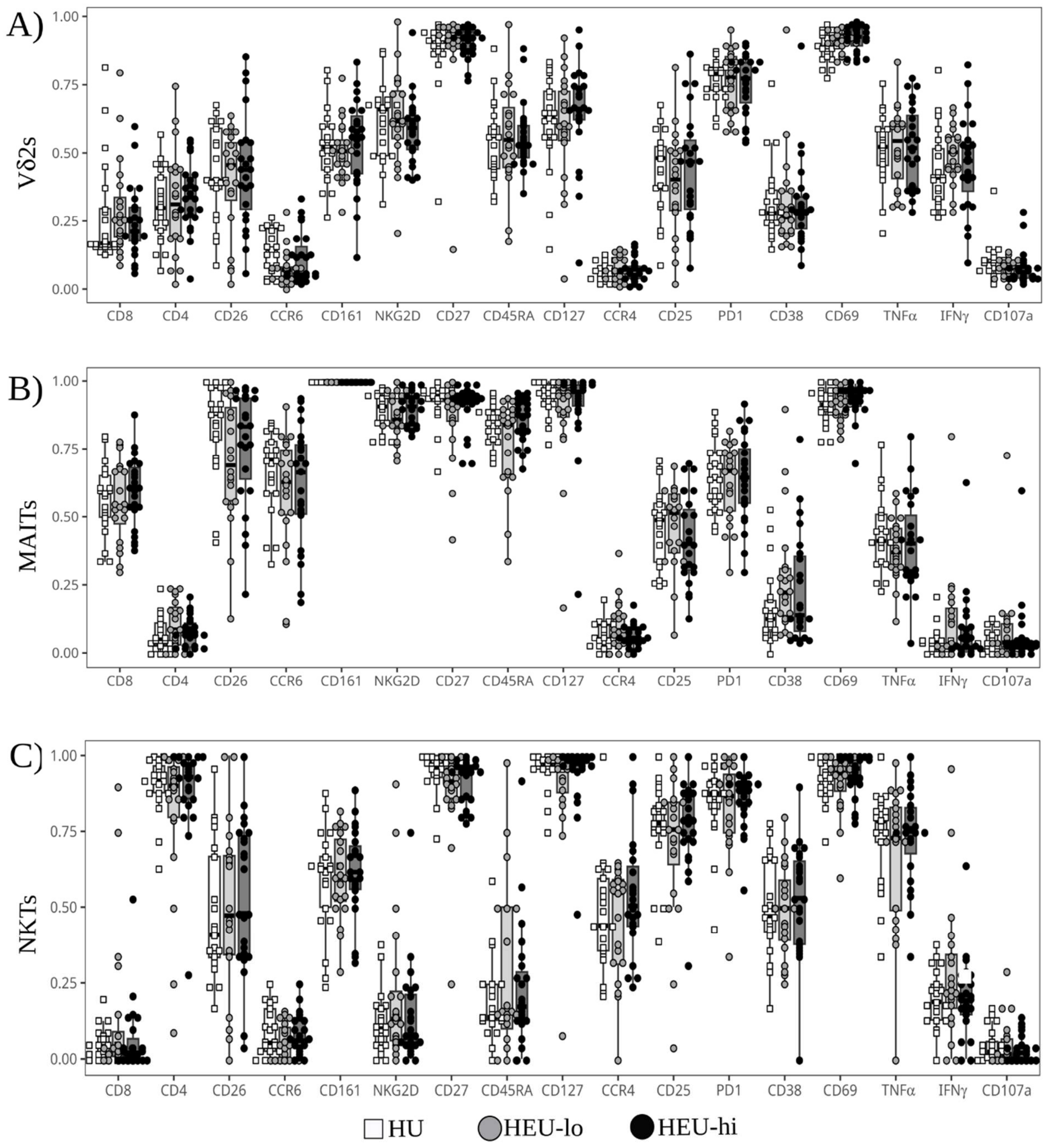

Supplemental Figure 9. Distinct functional profiles of cord blood ILT following polyclonal stimulation. (A) The dotplots of a representative specimen obtained from a HU neonate show the intracellular cytokine response (% of IFNγ+ by % of TNFα+) for Vδ2s cells (top), MAITs (middle), NKTs (bottom). (B-D) The beeswarm plots show the proportion of cells expressing individual markers listed on the x-axis for Vδ2 (B), MAIT (C) and NKT (D) cells, respectively. The symbols show individual values, boxplots show median and IQR, with whiskers indicating the +/- 1.5 IQR range. Shape and color identify HIV-exposure status.

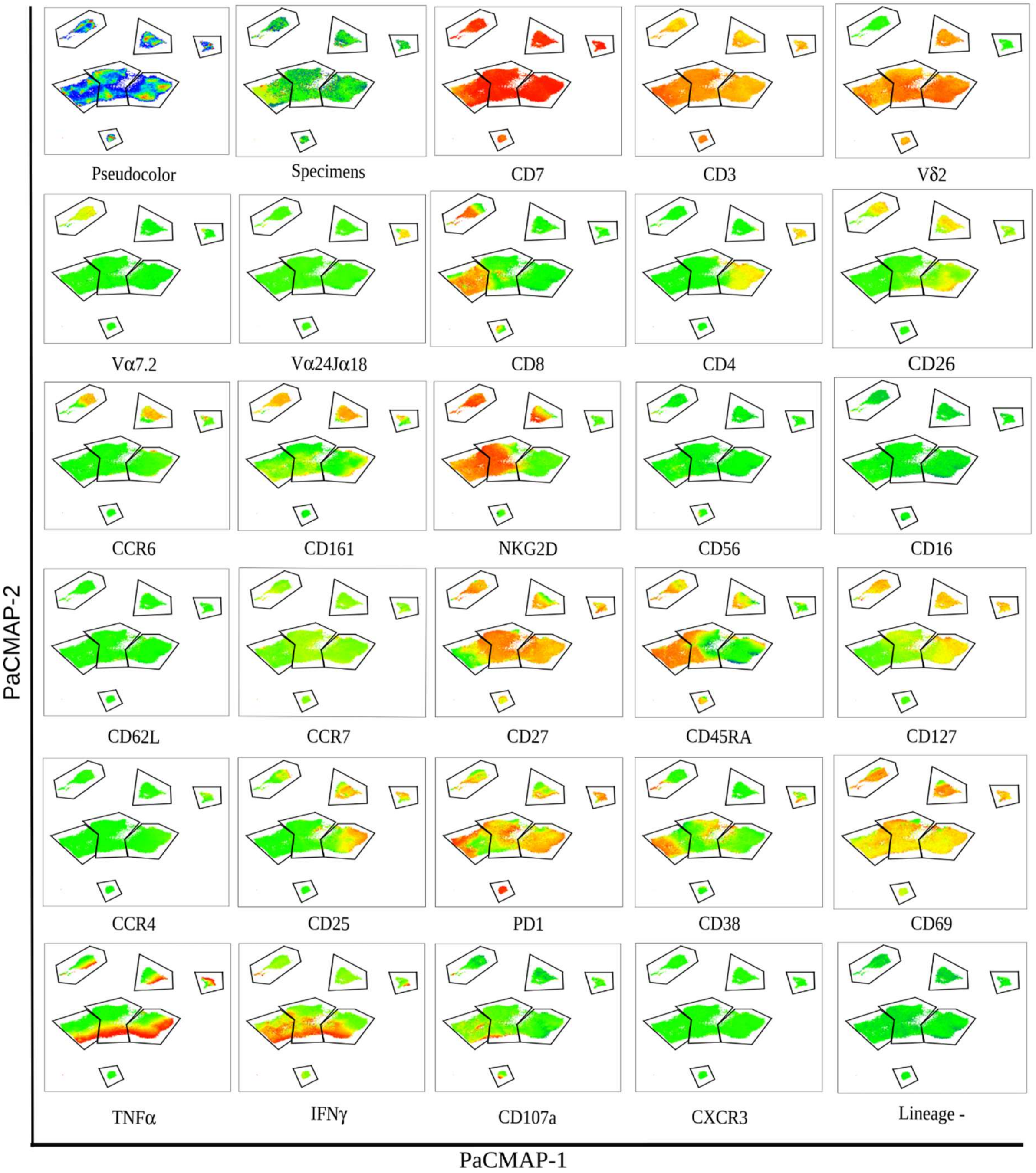

Supplementary Figure 10. Heatmap visualization for individual markers following polyclonal stimulation. Each plot in the grid shows the expression of individual markers by heat indexing on the basis of median fluorescent intensity (MFI) for PaCMAP generated ILT subsets in a concatenated file including Vδ2 cells and antibody identified MAITs and NKTs. Scale: green=low, red=high.

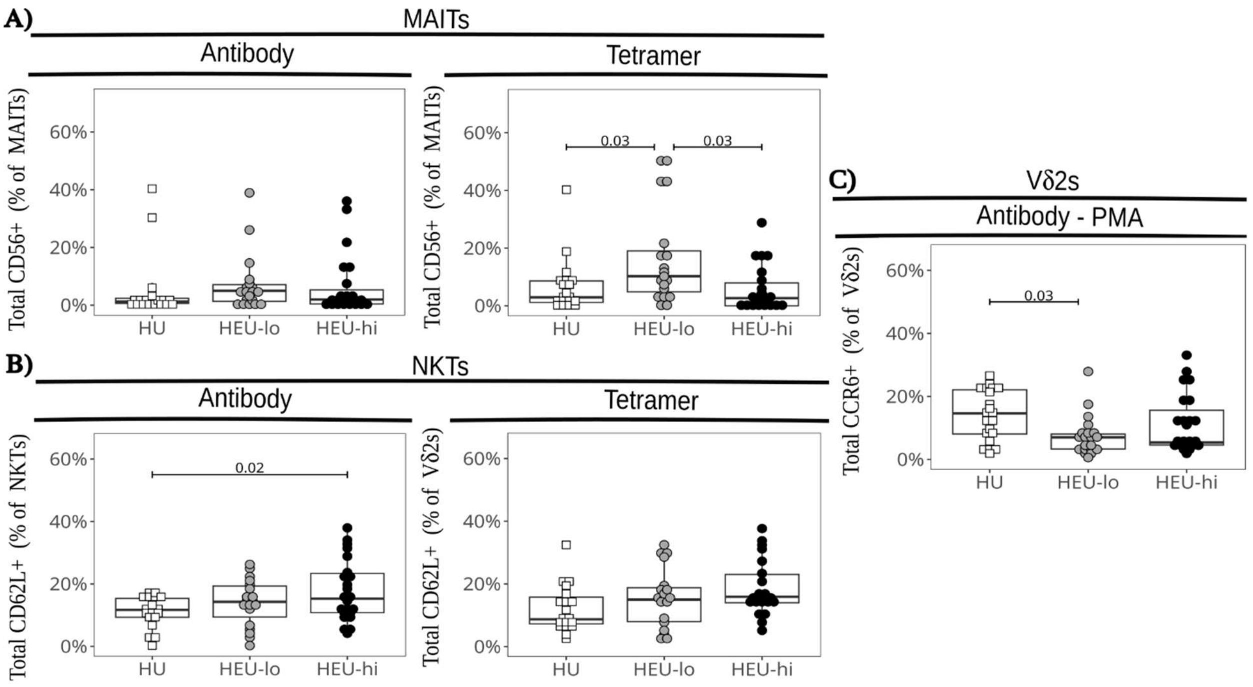

Supplementary Figure 11. Altered global marker expression in neonatal HEU ILT subsets. The beeswarm plots compare the expression of specific markers between exposure groups. Symbols show individual values, boxplots show median with IQR, and whiskers showing the +/- 1.5 IQR range. Shape and color identify HIV-exposure status. (A) Baseline proportion of CD56 expression for MAITs identified by antibody (CD161^{hi} Vα7.2+, left) or tetramer (CD161^{hi} hMR1 5-OP-RU+, right) staining. (B) Baseline proportion of total CD62L expression for NKTs identified by antibody (Vα24Jα18+, left) or tetramer (hCD1d PBS-57+, right) staining. (C) Frequency of CCR6+ Vδ2 cells after activation with PMA+ionomycin.

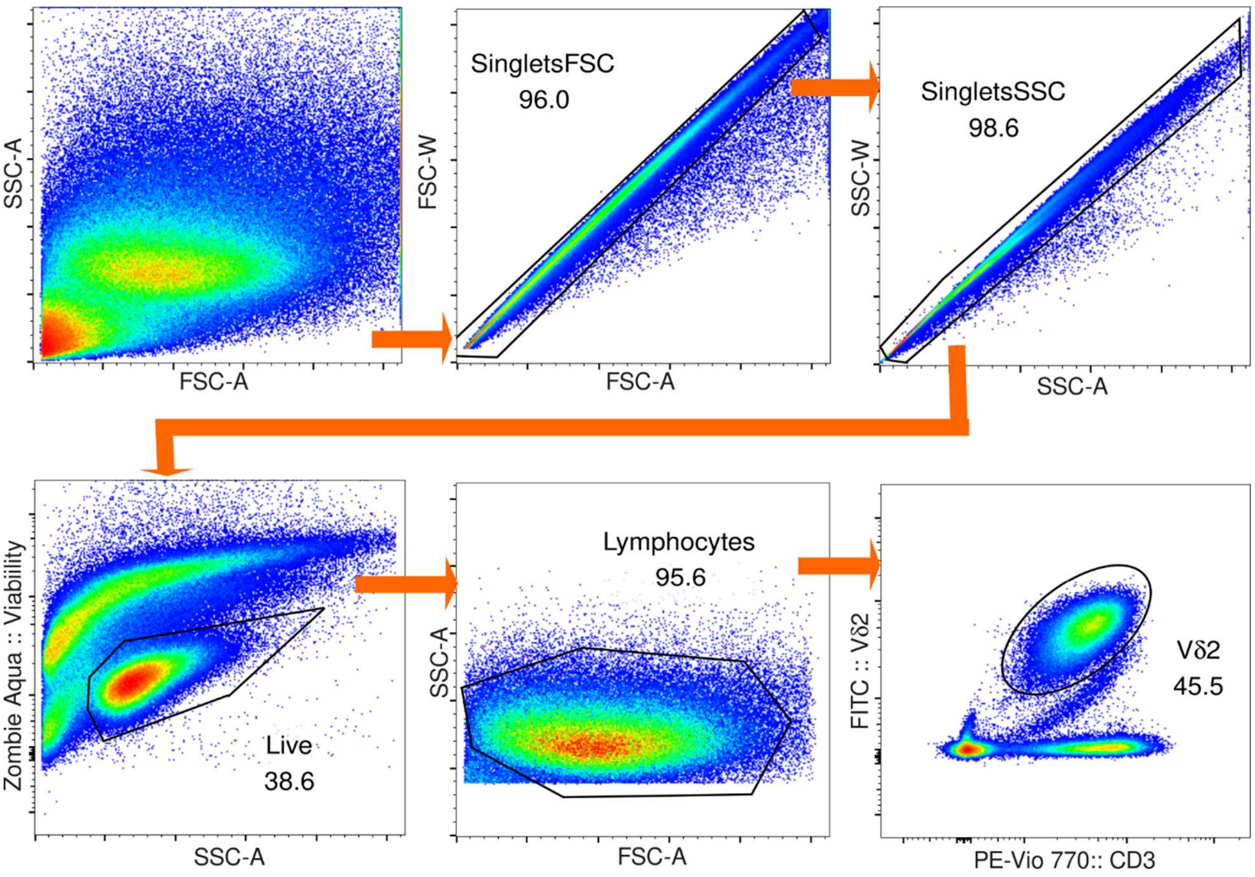

Supplementary Figure 12. Gating strategy for cultured day 17 conventional flow cytometry (CFC) panel. The dot plots illustrate the gating strategy used to identify expanded Vδ2 cells (D17) for a representative HU infant specimen, with descending hierarchical gates denoted by arrows. Individual gates and frequency in the parent gate are shown.

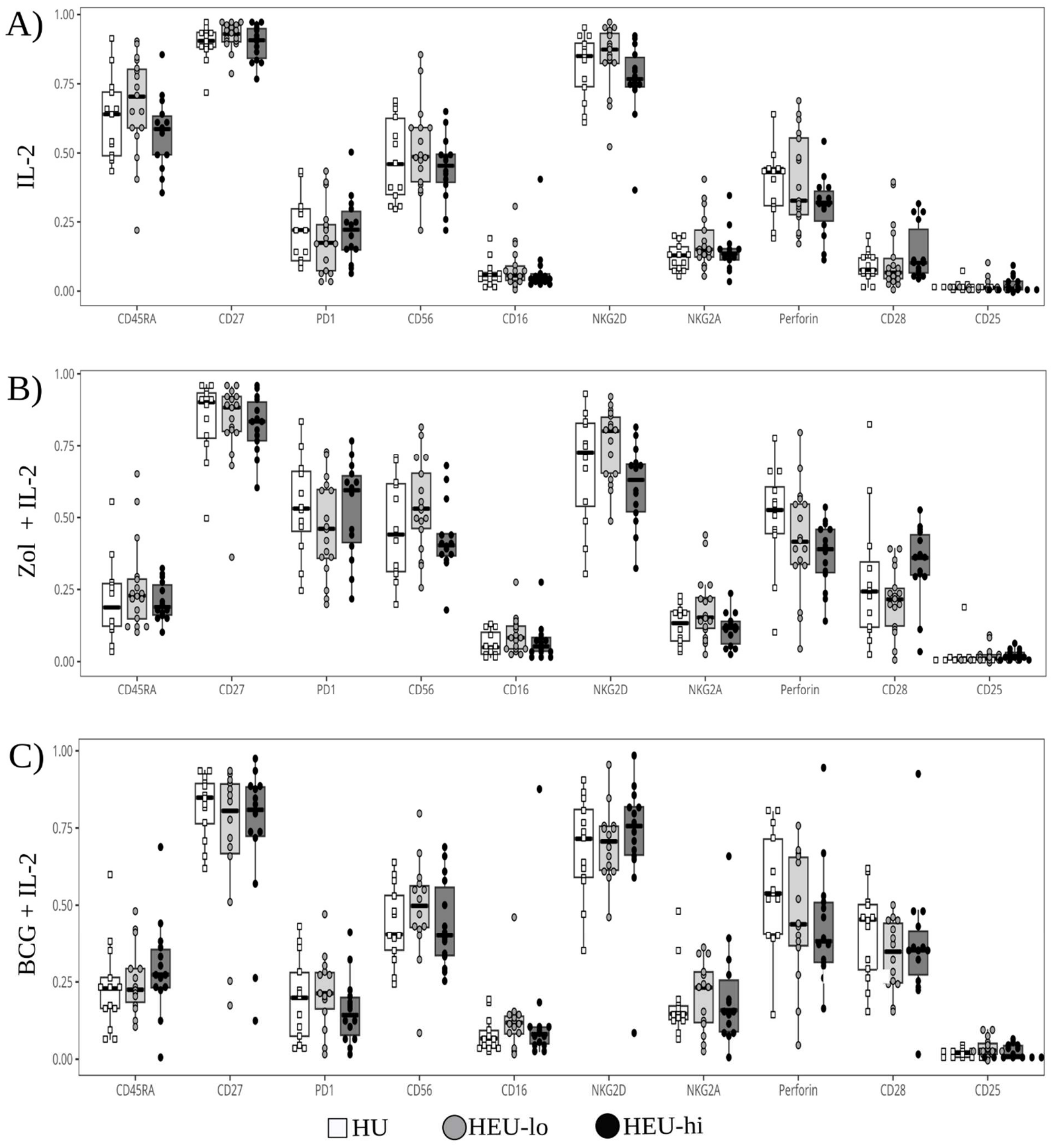

Supplementary Figure 13. Marker expression profiles of resting cord blood Vδ2 cells post-expansion. Thawed PBMC were stimulated with Zoledronate and IL-2, BCG and IL-2, or IL-2 alone, and expanded *in vitro* for 17 days. The beeswarm plots show the proportion of cord blood Vδ2 cells expressing individual markers listed on the x-axis after treatment with (A) IL-2 alone, (B) IL-2 and Zoledronate, and (C) IL-2 and BCG. The symbols show individual values, boxplots show median with IQR, and whiskers indicating the +/- 1.5 IQR range. Shape and color identify HIV-exposure status.

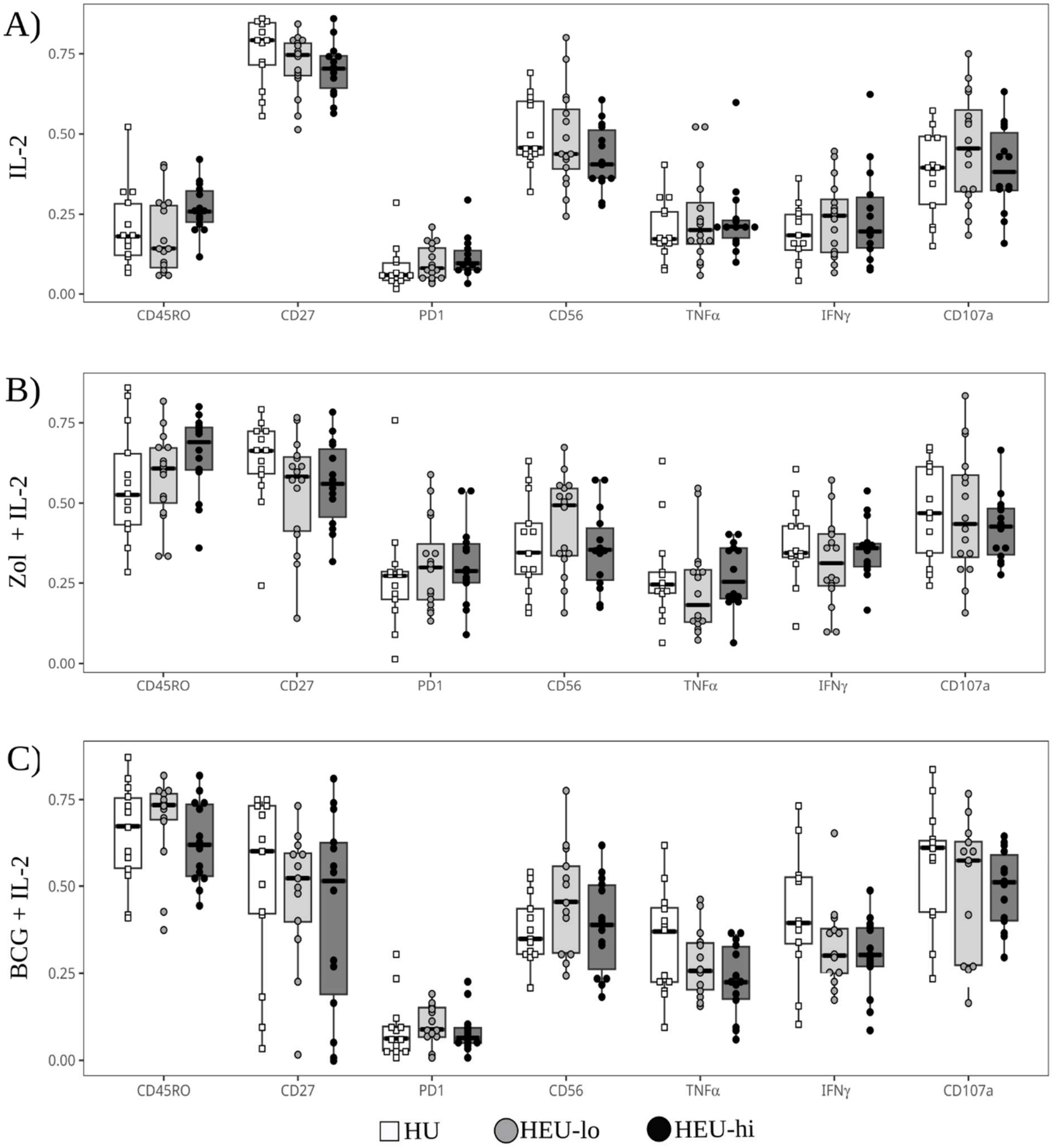

Supplementary Figure 14. Marker expression profiles of expanded cord blood Vδ2 cells after TCR-mediated restimulation. The beeswarm plots show the proportion of cells expressing the individual markers listed on the x-axis following reactivation with plate-bound anti-$\gamma\delta$ TCR for cord blood Vδ2 cells cultured for 17 days in the presence of (A) IL-2 alone, (B) IL-2 and Zoledronate, and (C) IL-2 and BCG. Symbols show individual values, boxplots show median and IQR, with whiskers indicating the +/- 1.5 IQR range. Shape and color identify HIV-exposure status.

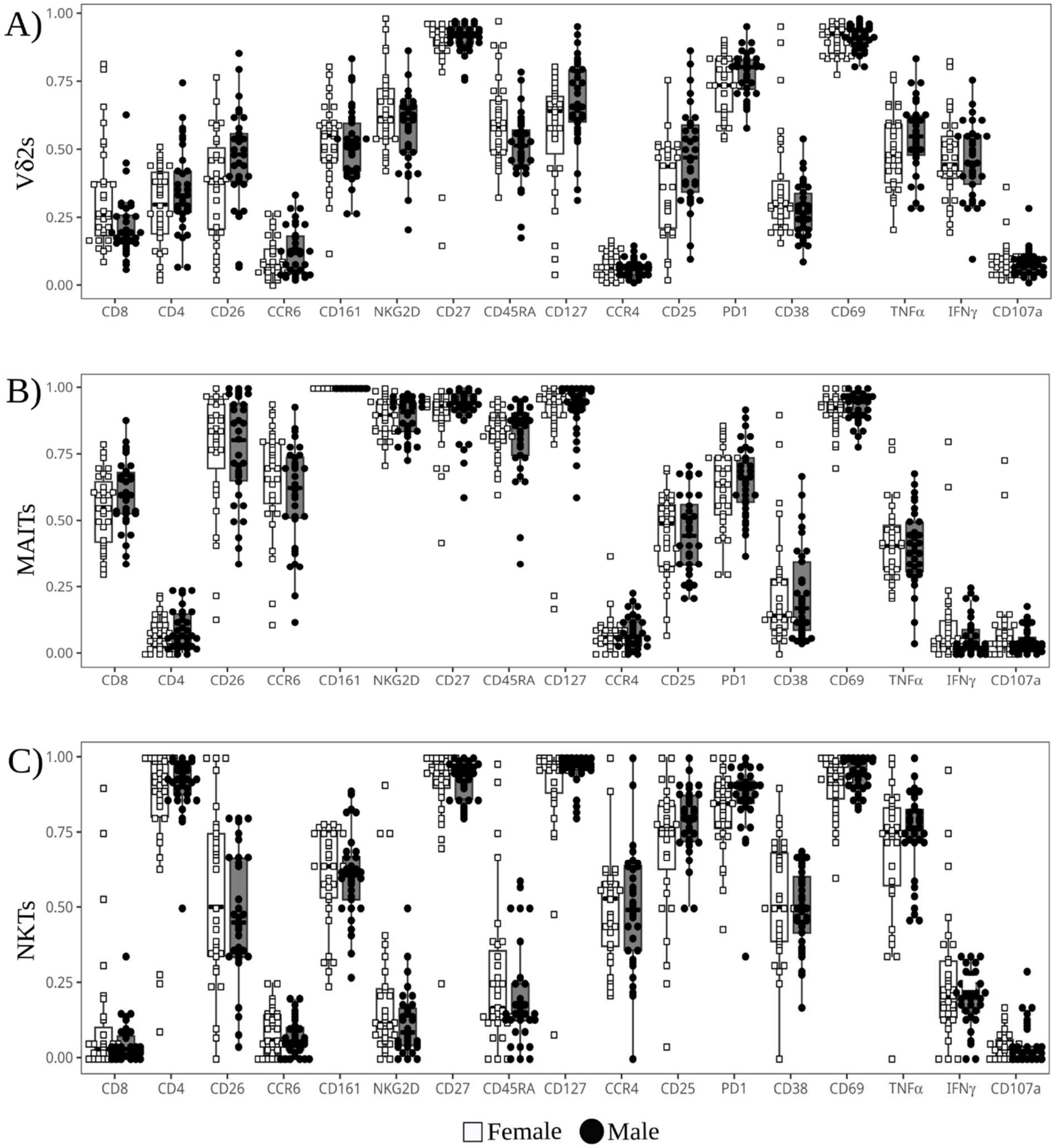

Supplementary Figure 15. Sex-based differences in ILT marker expression after polyclonal stimulation. (A) The beeswarm plots show the proportions of cells expressing individual markers listed on the x-axis for Vδ2 (A), MAIT (B) and NKT (C) cells, respectively. The symbols show individual values, boxplots show median and IQR, with whiskers indicating the +/- 1.5 IQR range. Shape and color identify infant sex.
